# Comparative Genomic Analysis of Quantitative Trait Loci Associated With Micronutrient Contents, Grain Quality, and Agronomic Traits in Wheat (*Triticum aestivum* L.)

**DOI:** 10.3389/fpls.2021.709817

**Published:** 2021-10-12

**Authors:** Nikwan Shariatipour, Bahram Heidari, Ahmad Tahmasebi, Christopher Richards

**Affiliations:** ^1^Department of Plant Production and Genetics, School of Agriculture, Shiraz University, Shiraz, Iran; ^2^USDA ARS National Laboratory for Genetic Resources Preservation, Fort Collins, CO, United States

**Keywords:** meta QTL analysis, comparative genomics, iron, zinc, candidate gene, GWAS

## Abstract

Comparative genomics and meta-quantitative trait loci (MQTLs) analysis are important tools for the identification of reliable and stable QTLs and functional genes controlling quantitative traits. We conducted a meta-analysis to identify the most stable QTLs for grain yield (GY), grain quality traits, and micronutrient contents in wheat. A total of 735 QTLs retrieved from 27 independent mapping populations reported in the last 13 years were used for the meta-analysis. The results showed that 449 QTLs were successfully projected onto the genetic consensus map which condensed to 100 MQTLs distributed on wheat chromosomes. This consolidation of MQTLs resulted in a three-fold reduction in the confidence interval (CI) compared with the CI for the initial QTLs. Projection of QTLs revealed that the majority of QTLs and MQTLs were in the non-telomeric regions of chromosomes. The majority of micronutrient MQTLs were located on the A and D genomes. The QTLs of thousand kernel weight (TKW) were frequently associated with QTLs for GY and grain protein content (GPC) with co-localization occurring at 55 and 63%, respectively. The co- localization of QTLs for GY and grain Fe was found to be 52% and for QTLs of grain Fe and Zn, it was found to be 66%. The genomic collinearity within Poaceae allowed us to identify 16 orthologous MQTLs (OrMQTLs) in wheat, rice, and maize. Annotation of promising candidate genes (CGs) located in the genomic intervals of the stable MQTLs indicated that several CGs (e.g., *TraesCS2A02G141400, TraesCS3B02G040900, TraesCS4D02G323700, TraesCS3B02G077100*, and *TraesCS4D02G290900*) had effects on micronutrients contents, yield, and yield-related traits. The mapping refinements leading to the identification of these CGs provide an opportunity to understand the genetic mechanisms driving quantitative variation for these traits and apply this information for crop improvement programs.

## Introduction

Most agricultural systems focus on increasing crop productivity and grain yield (GY) and fewer efforts have been devoted to the grain yield–quality tradeoff. However, a shift from prioritizing yield to more emphasis on quality, such as nutrient content is gaining ground in breeding programs (Khush et al., 2012). Extending the existing concepts for a simultaneous selection of GY, quality traits, and micronutrient contents seems necessary to facilitate the development of varieties with an effective combination of yield potential and end-use quality (Michel et al., 2019). A rapid increase in micronutrient deficiency in food grains has resulted in micronutrient malnutrition among consumers. Fe and Zn deficiencies are serious and prevalent sources of malnutrition in developing countries with high consumption of cereals, such as wheat (Black et al., 2013; Shahzad et al., 2014; Kumar et al., 2019). Diets based on staple food crops with Zn and Fe deficiency have been widely recognized as a major global health problem that affects almost three billion people (Murray and Lopez, 2013). Breeding crops through biofortification is a practical approach to cope with Fe and Zn deficiencies by increasing the grain Fe content (GFeC) and grain Zn content (GZnC) within the edible parts of staple food crops, especially cereals (Stein, 2010; Liu et al., 2019; Shariatipour and Heidari, 2020).

Wheat biofortification through breeding methods is a promising strategy to ameliorate Fe and Zn deficiencies in developing countries (Liu et al., 2019). One of the most important challenges in breeding for micronutrients are negative genetic trade-offs between yield and micronutrient traits (Flatt and Heyland, 2011; Fabian and Flatt, 2012). At the genetic level, such trade-offs are thought to be caused by alleles with antagonistic pleiotropic effects or by linkage disequilibrium between loci (Fabian and Flatt, 2012). While GFeC and GZnC biofortification is an important objective in wheat breeding programs, other important traits, such as GY and grain protein content (GPC) typically cannot be compromised. The wheat quality is measured by its rheological traits, such as GPC (Goel et al., 2019) since wheat is a major source of protein accounting for 19% of human protein intake in the developing countries (Braun et al., 2010). The genetic control of quality traits and GY is complex (Zilic et al., 2011; Velu et al., 2018; Giancaspro et al., 2019; Liu et al., 2019). A high priority of breeders is to develop high-quality genotypes that balance acceptable yield potential while maintaining quality characteristics, both of which are highly dependent on the co-variance between GY and the major quality (i.e., GZnC, GFeC, and GPC) criteria (Michel et al., 2019). However, a negative correlation between the GY and quality traits is challenging (Simmonds, 1995; Velu et al., 2016; Michel et al., 2019). In addition to this, our research indicates that protein concentration in grain decreases under elevated air CO_2_ concentrations of 550 μmol/mol (Fernando et al., 2012). By the end of the twenty-first century, it is predicted that the global temperature will rise from 1.1 to −3.1°C and the atmospheric CO_2_ concentration will reach above 550 ppm under intermediate scenarios (Pachauri et al., 2014) and that heat waves will occur with a higher frequency and longer duration (Pachauri et al., 2014). Given these future climate scenarios, it is critical to anticipate the effects of future growing environments and focus on breeding strategies that compensate for changes in grain quality. Likewise, it is important to have an inclusive breeding objective that tracks a portfolio of indirect traits responsible for grain quality and productivity, such as the assessment of dry matter accumulation, photosynthesis, coleoptile growth, carbon isotope discrimination, plant senescence, and rheological properties (Rebetzke et al., 2008; Liang et al., 2010; Vijayalakshmi et al., 2010; Goel et al., 2019).

Because of the large genome size and limited genome sequence information in wheat, the typical mapping intervals are quite large in most studies especially for the complex quality traits (Li Q. et al., 2020) and so further refinement is needed to narrow down the QTL intervals. Developing a statistically derived catalog of relevant loci is critical for developing marker-assisted selection (MAS) approaches in breeding programs. These markers can be applied to the quantitative trait loci (QTLs) that regulate the accumulation of high mineral nutrient concentration in grain along with QTLs for GY and grain quality traits. The QTL mapping method involves creating a QTL continuity map to identify genomic regions associated with quantitative traits (Mohan et al., 1997). Although QTL mapping is a powerful approach for detecting the genomic regions associated with complex traits, the genetic effects of QTL identified in different studies may not be present or are simply not tested in different genetic backgrounds and environments (Zhang L. Y. et al., 2010). In addition, the number of traits that can be measured in any single study is always resource limited (Acuña-Galindo et al., 2015). Overall, biparental populations are strongly influenced by different factors consisting of parents, the size and type of population, the choice of marker sets, and environmental conditions (Li et al., 2013; Izquierdo et al., 2018; Lei et al., 2018; Zhao et al., 2018).

In the last decade, an efficient approach called meta-QTL (MQTL) analysis has emerged in order to circumvent these restrictions. The MQTL method was initially developed by Goffinet and Gerber (2000) and was then improved by Veyrieras et al. (2007) is a method that gathers QTL data from independent experiments, years, location, and genetic backgrounds to detect stable QTLs (Goffinet and Gerber, 2000; Arcade et al., 2004; Hanocq et al., 2007; Sosnowski et al., 2012). The meta-QTL analysis integrates the information of QTLs from different population types and sizes identified in different environmental conditions to find stable MQTLs in a narrower genomic region with small CI (Goffinet and Gerber, 2000; Hanocq et al., 2007; Li et al., 2013).

The MQTL analysis allows for the dissection of genetic correlation among different traits (Truntzler et al., 2010; Danan et al., 2011; Xiang et al., 2012; Badji et al., 2018; Delfino et al., 2019). Hence, the MQTL analysis helps to enquire co-location of QTLs relying on dense marker maps that are responsible for different desirable traits including micronutrient contents, GY, and quality traits (Delfino et al., 2019). Currently, the MQTL analysis has become popular research in most studies (Goffinet and Gerber, 2000) related to micronutrients content (Raza et al., 2019), yield (Zhang L. Y. et al., 2010; Avni et al., 2018), and quality traits (Quraishi et al., 2017) to overcome the inconsistent QTL information reported for GZnC, GFeC, GPC, and yield traits. Further, MQTL analysis helps to identify the candidate genes (CGs) and refine genomic regions for yield and quality traits (Raza et al., 2019). However, little is known about the relation of micronutrients and quality-related MQTLs with agronomic traits in wheat.

The transferability of QTLs between cereals based on the analysis of syntenic regions and genomic collinearity helps to identify stable and important QTLs for use in breeding programs. The aims of this study were to perform a QTL meta-analysis to (1) identify QTLs that are consistently associated with grain quality, yield traits, and micronutrient content (2) explore the co-localized QTLs controlling GY, GPC, GZnC, and GFeC in the wheat genome, and (3) assess the transferability of QTLs between wheat, rice, and maize based on the comparative genomics and the orthologous MQTL (OrMQTS) mining. The outcome of this study will aid plant breeders in refining micronutrients, GY, and quality traits for crop improvement through marker-assisted breeding. Refining our understanding of the genetic architecture of micronutrients, GY, and quality traits often leads to the interrelationship between the regions of the genome that may be more challenging to breed independently. The MQTL is an analytic procedure that helps refine these relationships between CGs by providing for precision and statistical power.

## Materials and Methods

### QTL Database Development

A database consisted of 735 quantitative trait loci (QTLs) derived from 27 independent mapping populations (assessed between 2006 and 2019) assigned to 70 traits (Table 1) was used for the meta-QTL (MQTL) analysis. The independent populations consisted of 20 recombinant inbred lines (RILs) and 7 double haploids (DH) populations, with population sizes ranging from 92 to 485 lines (Supplementary Table 1). The reported position, the proportion of the phenotypic variance (*R*^2^), and the logarithm of odds (LOD score) of the initial QTLs were used for the analysis of meta-QTLs. For QTLs with missing LOD or *R*^2^, the values were estimated by the following equation (Nagelkerke, 1991):


$$\begin{array}{l} {\text{R}}^{2}=1-10^{\left(-2\text{LOD}/\text{n}\right)} \end{array}$$

where n represents the size of the population.

**Table 1:** The list of assessed traits in meta-quantitative trait loci (MQTL) analysis.

**Trait**	**Abbreviation**	**Trait**	**Abbreviation**
200KW	200-kernel weight	GY	Grain yield
25%G	25% green leaf area	GZnC	Grain Zn content
50%G	50% green leaf area	HI	Harvest index
75%G	75% green leaf area	HW	Hectoliter weight
AGB	Above ground biomass	KH	Kernel hardness
BDT	Break down time	KL	Kernel length
BM	Biomass	KW	Kernel width
BY	Biological yield	LDMA	Leaves dry matter accumulation
CDMA	Culm dry matter accumulation	LL	Leaf length
CID	Carbon isotope discrimination	LS	Lodging score
DA	Days to anthesis	LW	Leaf width
DDT	Dough development time	LY	Leaf yellowing
DGC	Dry gluten content	MDR	Maturity date
DH	Days to heading	MRS	Maximum rate of senescence
DPM	Days to physiological maturity	MTI	Mixing tolerance index
DST	Dough stability time	NG	Number of grain per spike
DTF	Days to flowering	PDMA	Plants dry matter accumulation
FFD	Factor form density	PGMS	Percent green at maximum senescence
FLH	Flag leaf height	PH	Plant height
FWA	Flour water absorption	PLH	Penultimate leaf height
GAS	Grain area size	PT	Productive tillers/m^2^
GCuC	Grain Cu content	SD	Seed diameter
GFD	Grain filling duration	SDS	Sedimentation rate
GFeC	Grain Fe content	SHS	Shattering score
GFR	Grain filling rate	SHZnC	Shoot Zn content
GL	Grain length	SL	Spike length
GL/GW	Grain length/grain width ratio	SN	Spike number
GMnC	Grain Mn content	SNS	Spikelet number per spike
GN	Grain number	SW	Spike weight
GPC	Grain protein content	TKW	Thousand kernel weight
GPL	Grain perimeter length	TMRS	Time to maximum rate of senescence
GSeC	Grain Se content	TN	Tiller number/m^2^
GW	Grain width	UIH	Uppermost internode height
GWe	Grain weight/ear	WGC	Wet gluten content
GWs	Grain weight/spike	ZnE	Zn efficiency

### Constructing Consensus Genetic Map and QTL Projection

The data files of the 27 maps were integrated with the Somers (Somers et al., 2004) reference map for the construction of a consensus genetic map. Attempts to use other mapping studies consisting of single-nucleotide polymorphism (SNP) were unsuccessful due to the lack of SNP density in the regions for the meta-QTL analysis. The constructed map file for each population consisted of information on cross-type, population size, map function, map units, and the position of different markers in different linkage groups. The individual QTLs derived from independent populations were projected onto the consensus genetic map consisted of 3,394 markers with a total length of 3,412.5 cM.

### Meta-QTL Analysis

Meta-QTL analysis was performed in BioMercator v4.2 (Arcade et al., 2004; Sosnowski et al., 2012). For n QTLs, the BioMercator tests the most likely assumption based on Akaike information criterion (AIC), corrected AIC (AICc), AIC 3 candidate models (AIC3), Bayesian information criterion (BIC), and an average weight of evidence (AWE) criteria in which the prevalent value among them was considered as the best fit. The consensus QTL from the optimal model was reported as MQTL. Consequently, the MQTL position and distribution on each linkage group were presented as a heatmap using *pheatmap* R package (Kolde, 2013). Moreover, the initial QTLs with 95% confidence interval (CI), QTL density in the identified MQTL, and the distribution of MQTLs were drawn on the linkage groups using shinyCircos web tool based on the R program (Yu et al., 2017). The variation of QTL density for different traits toward centromeric and telomeric genomic regions was estimated following the approach by Martinez et al. (2016). The QTL density was determined by counting the number of QTLs for each trait on 50 cM intervals across the wheat genome, starting from the centromere region of a linkage group at position 0. The centromere position was retrieved from the study by Wan et al. (2017).

### Functional Candidate Genes in MQTLs Intervals

The MQTLs containing more than five trait-QTLs from different experiments were considered as the most stable consensus regions and were analyzed for the detection of functional candidate genes (CGs). To identify the functional CGs, the sequences of the flanking markers for each MQTL were retrieved from “Grain Genes” database (https://wheat.pw.usda.gov/browse?class=probe;query=BARC%2A;begin=351) for the simple sequence repeat (SSR) flanking markers and “Diversity Array Technology” (https://www.diversityarrays.com/) (DArT) flanking markers. For flanking markers lacking a definite position on the wheat genome, the closest markers on the genetic consensus map were selected to determine the MQTL position. Additionally, for those flanking markers lacking sequence information in databases, the forward and reverse sequences were retrieved from the “Grain Genes” database and were used for the Basic Local Alignment Search Tool (BLAST) analysis against the newest wheat reference genome (IWGSC RefSeq v2.0) for detecting the genomic position of each MQTL. The annotation and gene ontology (GO) of genes lying at the MQTL interval were retrieved from EnsemblPlants (http://plants.ensembl.org/index.html) using the new wheat genome (IWGSC v2.0). Finally, the orthologous of genes located at each MQTL interval were investigated in rice to describe the functional CGs based on their reported functions in wheat or rice.

### Identification of Traits Within MQTLs

To analyze traits within the MQTL regions, the MQTL results were converted into binary scores (0 or 1) on the basis of the absence/presence of an individual trait-QTL within an MQTL region. We tabulated the number of times a trait was present within an MQTL, the number of QTLs for a trait present within an MQTL (implying confirmation of the QTL), and the number of times the traits were able to be co-localized within an MQTL. A chi-squared test with one degree of freedom was performed to determine traits showing significant co-localization with grain protein content (GPC), grain zinc content (GZnC), grain Fe content (GFeC), and grain yield (GY) beyond what would be expected for a random distribution of QTL within MQTL throughout the genome. The expected number of MQTL associated with a trait and each GPC, GZnC, GFeC, and GY were separately calculated by multiplying the number of observed MQTL for a trait by the proportion of MQTL containing GPC, GZnC, GFeC, and GY QTL(s). Traits within the MQTL regions were also analyzed using IBM SPSS Statistics v.24. The simple regression analysis was performed using Minitab v. 18 to determine the effect of MQTLs on the association of GY, GPC, GFeC, and GZnC.

### MQTLs and GWAS Comparison

The detected MQTLs were compared with the significant loci associated with different quantitative traits identified in wheat genome-wide association studies (GWAS). The mapped coordinates of the identified significant loci through GWAS were compared to those found with the MQTL analysis.

### Orthologous MQTL

Due to the high synteny among genes in Poacaea, the most stable and promising wheat MQTLs were evaluated for the detection of the orthologous MQTLs (OrMQTL) in rice (Lei et al., 2018; Raza et al., 2019; Khahani et al., 2020), and maize (Semagn et al., 2013; Wang et al., 2013, 2016). A set of orthologous genes at the MQTLs regions was considered as a criterion of a syntenic region using EnsemblPlants (http://plants.ensembl.org/index.htmldatabase).

## Results

### Genomic Quantitative Trait Loci Distributions

The individual traits of quantitative trait loci (QTLs) used in this study are listed in Supplementary Table 2. The QTLs for thousand kernel weight (TKW) (18.03%), grain yield (GY), (13.11%), and a number of grains per spike (NS) (13.11%) were the most frequently reported agronomic QTLs identified in the tested mapping populations. The grain Fe content (GFeC) (33.33 %) and grain Zn content (GZnC) (28.21 %) of QTLs for micronutrient traits and grain protein content (GPC) (64.00 %) and sudden death syndrome (SDS) (10.00 %) of QTLs for quality traits were frequent.

**Figure 1:**
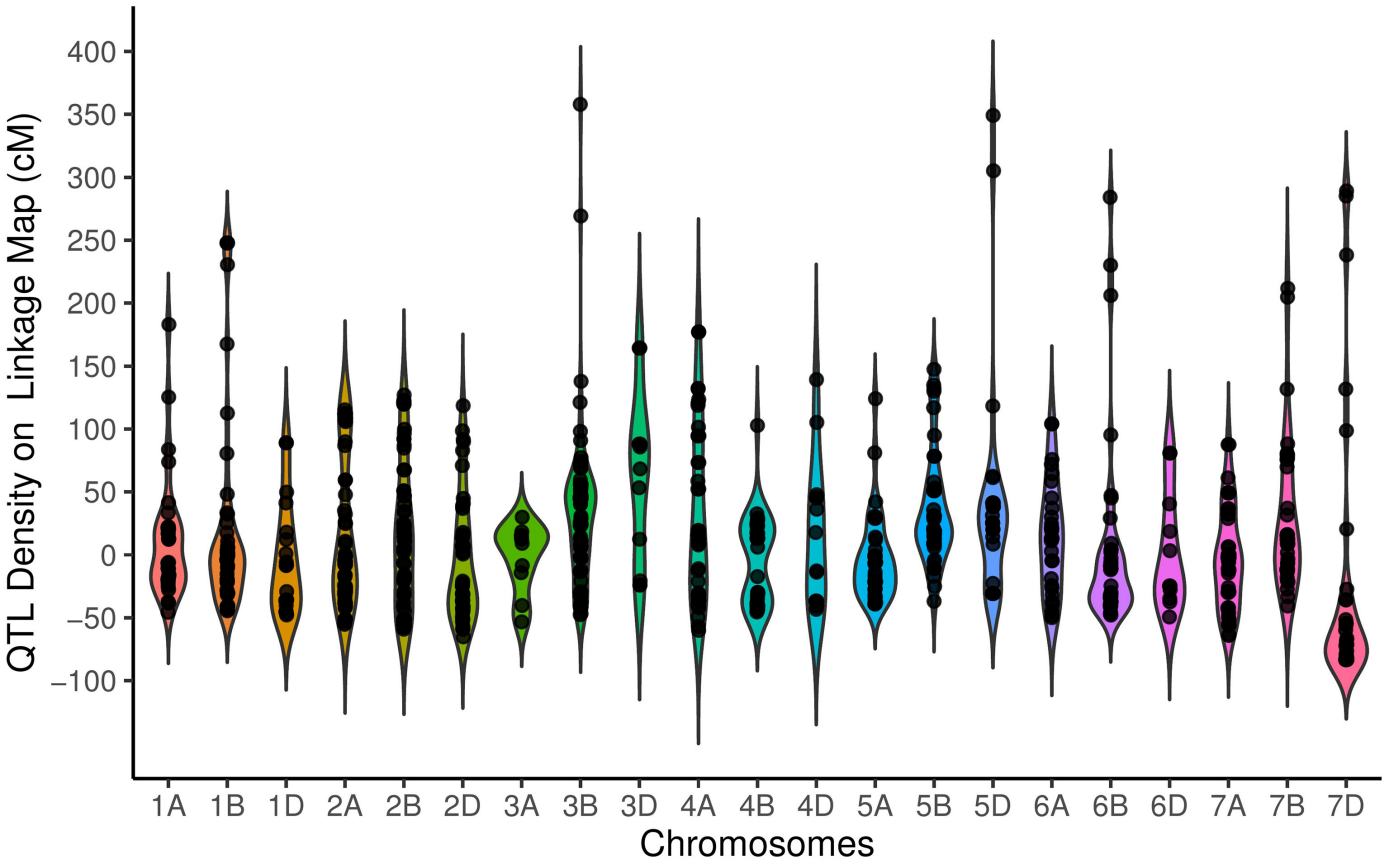
Distribution of quantitative trait loci (QTLs) for traits on all chromosomes represented as the number of QTLs per distance (50 cM), starting from the centromeric region of each chromosome where it was considered at the position 0 cM. Each dot represents the exact location of each QTL.

Our results suggest a non-random distribution of QTLs within the wheat genome. Distribution of QTLs on the basis of physical size [χ^2^_(2)_ = 60.17, *P* = 8.58E−14] showed that 254, 326, and 155 QTLs were located on the A, B, and D genomes, respectively. The QTL distribution was significantly different among the seven chromosome groups [$\chi_{\left(6\right)}^{2}$ = 47.12, *P* = 1.77E−8], ranging from as few as 76 QTLs on group 6 to as many as 165 QTLs on Group 2. Chromosome 3B with 75 QTLs had the highest number of QTLs, followed by chromosome 2B (62 QTLs) and 2A (61 QTLs), while chromosome 3A with 10 QTLs had the lowest QTL. The distribution of QTL over the genetic linkage map with respect to centromeric and telomeric regions was distinctly non-random (Figure 1). The non-telomeric region of each chromosome (−50 up to + 50 cM intervals) had the highest number of QTLs (Figure 1). We did not detect QTLs at 100 cM for the tested traits.

**Figure 2:**
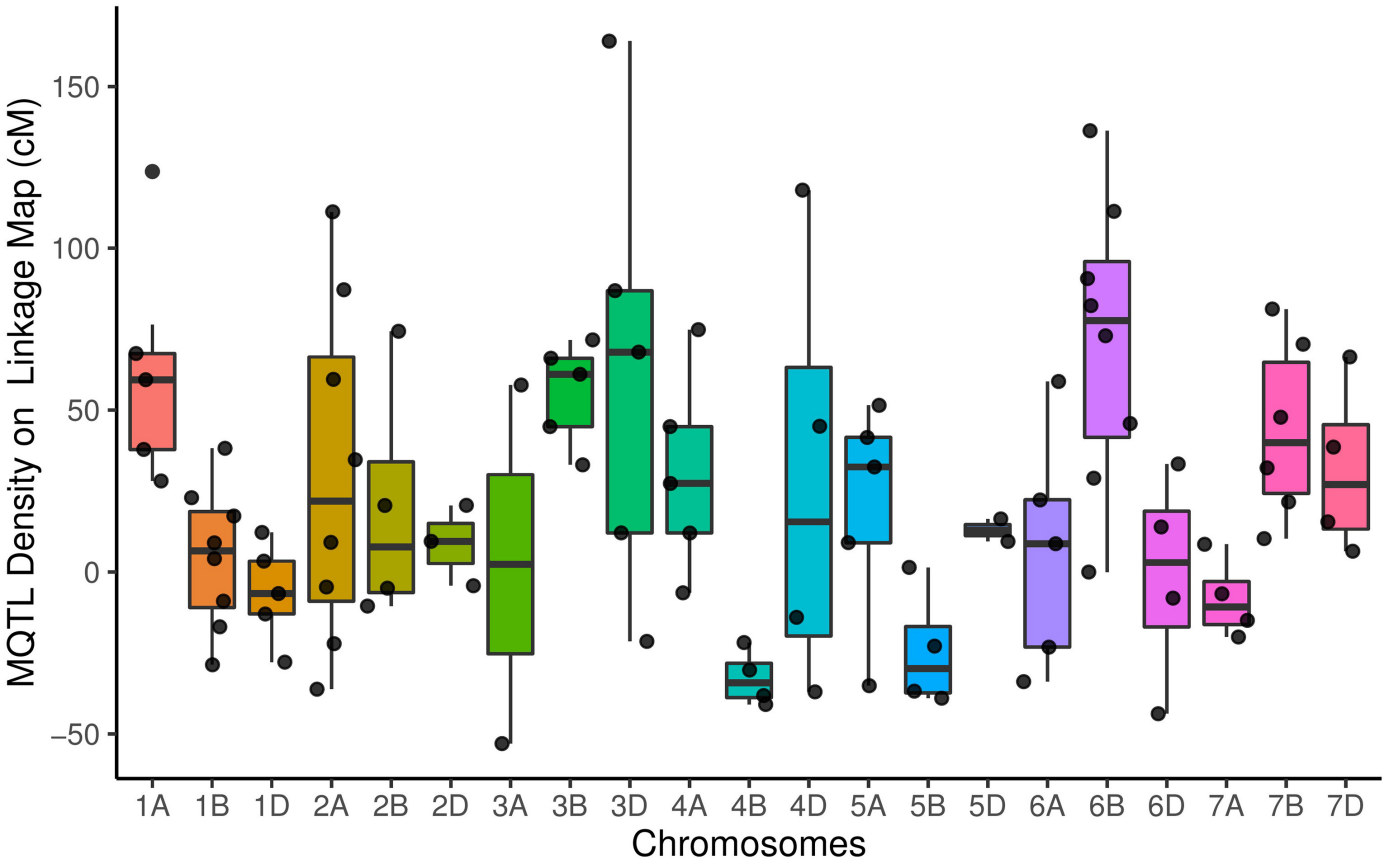
Distribution of meta-quantitative trait loci (MQTLs) for assessed traits on all chromosomes represented as a number of MQTLs per distance (50 cM), starting from the centromeric region of each chromosome where it was considered at position 0 cM. Each dot represents the exact location of each MQTL.

The distribution of meta QTLs (MQTLs) indicated that a cluster of MQTLs was mapped to the non-telomeric regions. Besides, MQTL_5B_4 and MQTL_6B_1 were located near the centromeric region of chromosomes 5B and 6B, respectively (Figure 2). There was a significant correlation between the number of initial QTLs and MQTLs (*r* = 0.46, *P* < 0.03). The number of MQTLs per chromosome varied from two (chromosomes 3A and 5D) to eight (chromosomes 1B, 2A, and 6B).

### Meta-QTL Analysis

**Figure 3:**
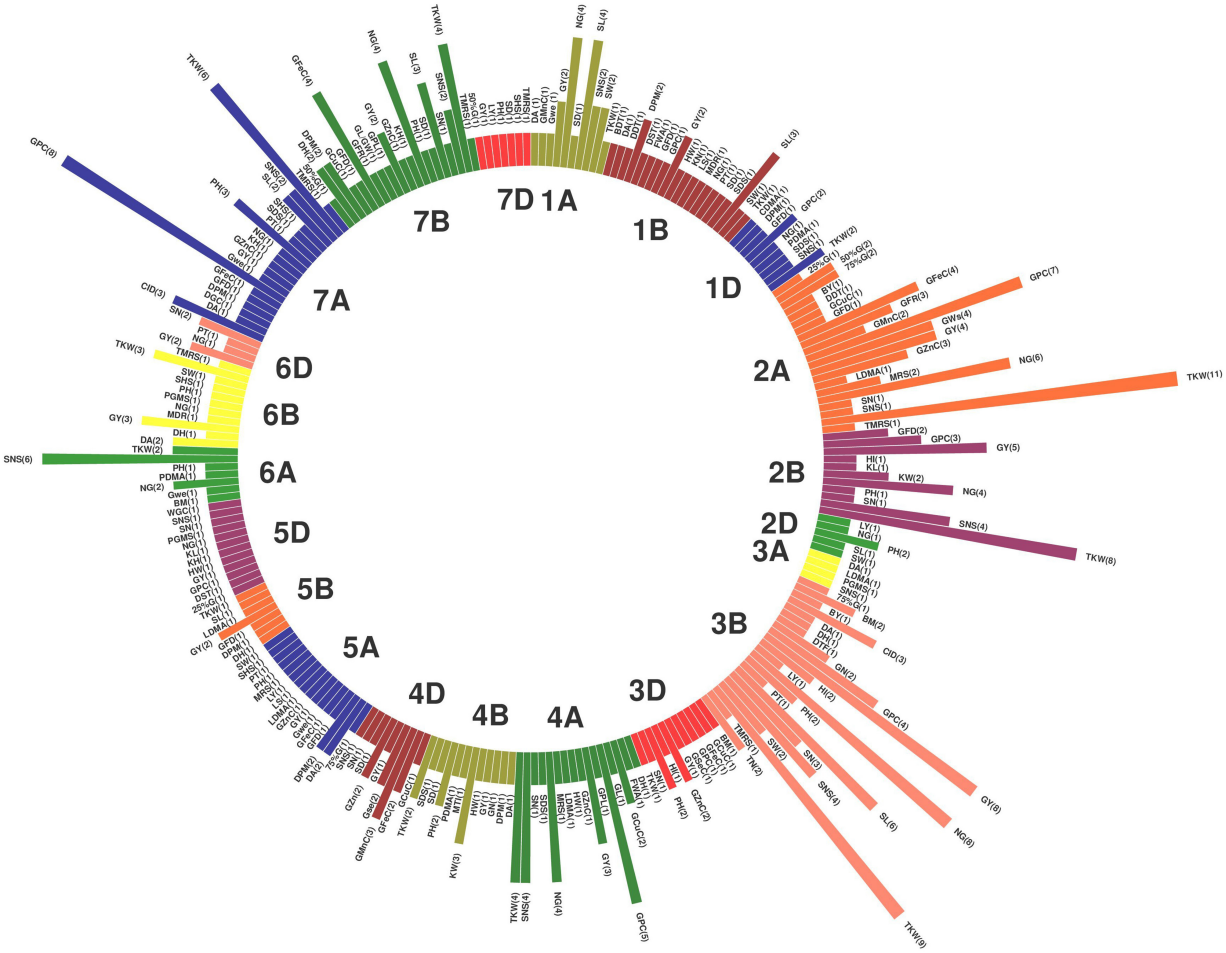
Distribution of projected quantitative trait loci (QTLs) across the wheat (*Triticum aestivum* L.) chromosomes. The numbers inside each parenthesis represent the number of QTLs.

**Figure 4:**
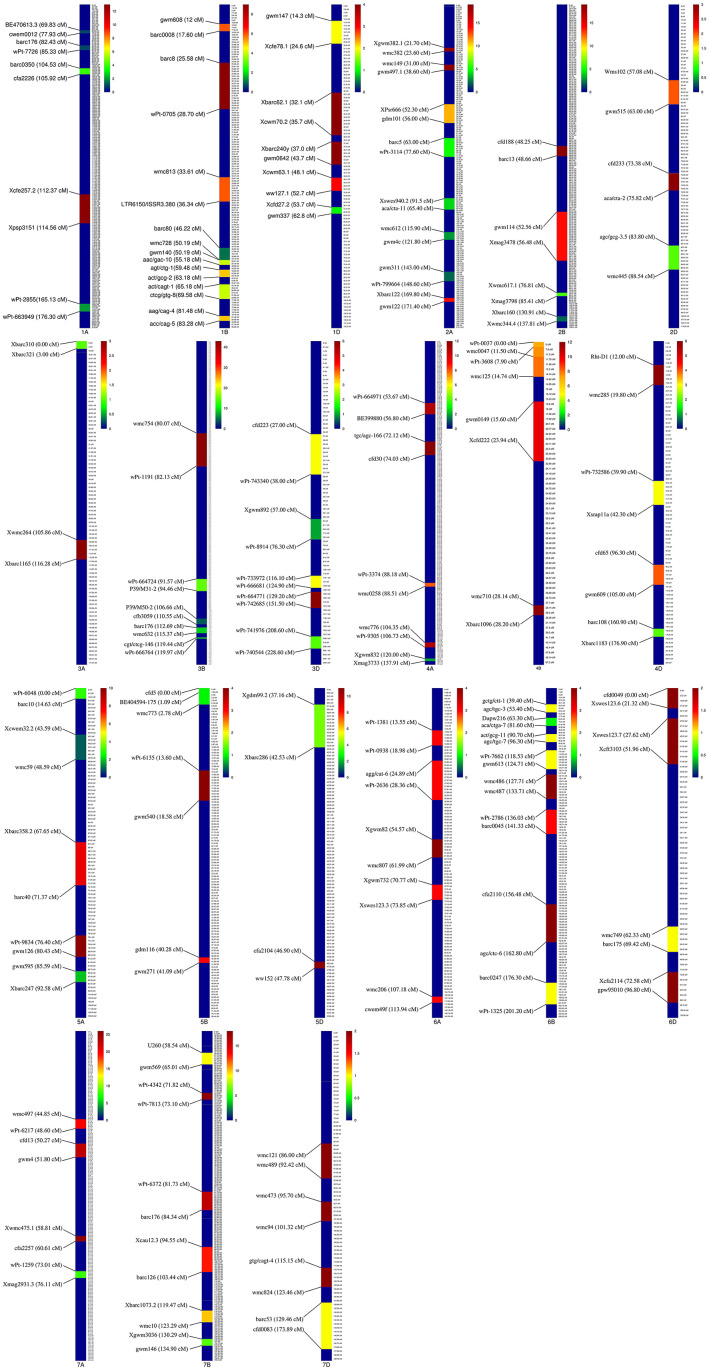
Position of detected meta-quantitative trait loci (MQTLs) on the wheat genome associated with micronutrient content, grain quality, and quantitative traits with 95% confidence interval (CI). Each color in a different linkage group indicates the number of initial QTLs involved in each MQTL. The flanking markers for each MQTL are presented on the left side of the linkage groups.

**Figure 5:**
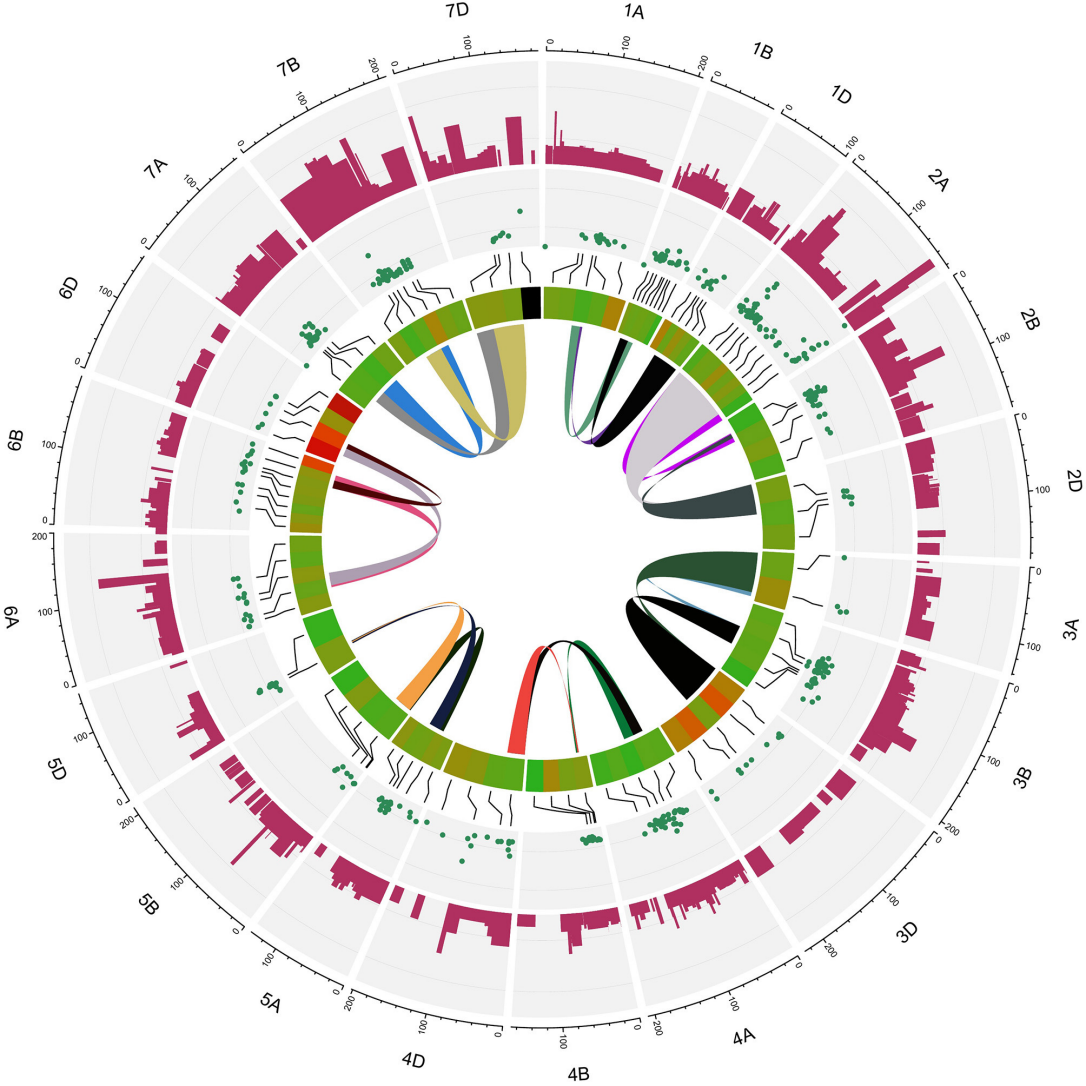
Circus plot showing distribution of QTLs and MQTLs on 21 linkage group of wheat. The outermost circle indicates the length of each linkage map on the consensus genetic map. The second circle indicates the initial QTLs with 95% confidence intervals. The third circle displays the density of QTLs in each MQTLs. The fourth circle displays the position of detected MQTL related to assessed traits based on heatmap illustration. The last inner circle demonstrated common markers links between different genome (A, B, and D) of each linkage groups.

Of the 735 initial QTLs, 449 were successfully projected onto the genetics consensus map and used in the meta-QTL analysis (Figure 3). A total of 100 MQTLs were detected and the number of individual QTL per MQTL ranged from 1 to 43 (Figures 4, 5; Supplementary Figure 1). The number of traits present per MQTL region ranged from 1 to 18. Among the identified MQTLs, MQTL_3B_1 that contained 43 QTLs had the highest number of initial QTLs followed by MQTL_7A_3 with 29 initial QTLs (Supplementary Table 3). These two MQTLs can be considered as the most stable QTLs under different experimental conditions. The detailed information of MQTLs consisted of the chromosome number, position, confidence interval (CI), flanking markers, and traits which are shown in Table 2. The higher marker density of the consensus map compared with the lower marker density in the independent linkage maps helped to reduce the CI of QTLs up to three-fold with an average of 4.63 cM in MQTLs compared with the mean CI of 13.73 cM for the original QTLs. Among the detected MQTLs, the CI of 11 MQTLs was reduced up to <1 cM (Table 2).

**Table 2:** Description of detected meta-quantitative trait loci (MQTL).

**MQTL name**	**Chromosome**	**Flanking markers**	**Position (cM)**	**CI (cM)**	Individual QTL present in MQTL^*^
MQTL_1A_1	1A	BE470613.3–cwem0012	74.13	3.6	GWe
MQTL_1A_2	1A	barc176–wPt-7726	83.88	2.9	SNS
MQTL_1A_3	1A	barc0350–cfa2226	105.4	1	GY, SD, SL, SNS, TKW
MQTL_1A_4	1A	Xcfe257.2–Xpsp3151	113.51	2.1	DA, GY, NG, SD, SL, SW, TKW
MQTL_1A_5	1A	wPt-2855–wPt-663949	169.72	8.6	GMnC, SL
MQTL_1B_1	1B	gwm608–barc0008	15.34	4.31	DA, DPM, GFD, HW, SD, SDS, TKW
MQTL_1B_2	1B	barc8–wPt-0705	27.06	2.98	BDT, DA, DPM, GFD, GPC, HW, SDS, SL, TKW
MQTL_1B_3	1B	wmc813–LTR6150/ISSR3.380	35.00	2.75	BDT, GFD, GY, HW, NG, SDS, TKW
MQTL_1B_4	1B	barc80–wmc728	48.11	3.2	BDT, DDT, DST, FWA
MQTL_1B_5	1B	gwm140–aac/gac-10	53.01	3.19	DDT, DST, FWA, GY
MQTL_1B_6	1B	agt/ctg-1–act/gcg-2	61.31	2.49	DDT, DST, FWA, LS, SW
MQTL_1B_7	1B	act/cagt-1–ctcg/gtg-8	66.96	2.39	DDT, DST, FWA, MDR
MQTL_1B_8	1B	aag/cag-4–acc/cag-5	82.21	0.42	DDT, DST, FWA, NG, SL
MQTL_1D_1	1D	gwm147–Xcfe78.1	19.18	9.09	GFD, GPC, SDS, SNS
MQTL_1D_2	1D	Xbarc62.1–Xcwm70.2	34.03	2.94	DPM, GPC, SDS, TKW
MQTL_1D_3	1D	Xbarc240y–gwm0642	40.35	6.75	CDMA, DPM, GPC, PDMA, SDS
MQTL_1D_4	1D	Xcwm63.1–ww127.1	50.36	4.12	NG, PDMA
MQTL_1D_5	1D	Xcfd27.2–gwm337	59.23	2.18	CDMA, PDMA, TKW
MQTL_2A_1	2A	Xgwm382.1–wmc382	22.80	1.4	50%G, 75%G, GFeC, GPC, GY, GZnC, MRS, SNS, TKW, TMRS
MQTL_2A_2	2A	wmc149–gwm497.1	36.87	3.35	50%G, 75%G, GCuC, GFeC, GMnC, GPC, GWs, GY, MRS, NG, SNS, TMRS
MQTL_2A_3	2A	XPsr666–gdm101	54.37	3.09	25%G, 50%G, 75%G, DDT, DTF, GFD, GWs, GY, MRS, NG, TKW
MQTL_2A_4	2A	barc5–wPt-3114	68.17	6.85	BY, GFD, GY, MRS, NG
MQTL_2A_5	2A	Xswes940.2–aca/cta-11	93.68	3.15	GFR, GPC, LDMA, TKW
MQTL_2A_6	2A	wmc612–gwm4c	118.53	4.94	GFR, SN, TKW
MQTL_2A_7	2A	gwm311–wPt-799664	146.19	3	GMnC, NG
MQTL_2A_8	2A	Xbarc122–gwm122	170.28	0.39	GFR, GWs, GY, NG, KW
MQTL_2B_1	2B	cfd188–barc13	48.48	0.42	GFD, GY, KL, KW, NG, PH, SNS, TKW
MQTL_2B_2	2B	gwm114 -Xmag3478	54.01	2.68	GY, KL, KW, NG, SN, SNS, TKW
MQTL_2B_3	2B	Xwmc617.1–Xmag3798	79.57	4.22	GPC, HI, SNS, TKW
MQTL_2B_4	2B	Xbarc160–Xwmc344.4	133.37	1.29	GY, NG
MQTL_2D_1	2D	wms102–gwm515	60.77	3.82	PH, SL, SW
MQTL_2D_2	2D	cfd233–aca/cta-2	74.48	1.97	NG, LY, PH
MQTL_2D_3	2D	agc/gcg-3.5–wmc445	85.6	3.58	PH
MQTL_3A_1	3A	Xbarc310–Xbarc321	1.00	3	LDMA
MQTL_3A_2	3A	Xwmc264–Xbarc1165	111.77	7	DA, PGMS, SNS
MQTL_3B_1	3B	wmc754–wPt-1191	81.11	1.99	BM, CID, DA, DTF, GN, GPC, GY, HI, LY, NG, PH, SL, SN, SNS, SW, TKW, TN
MQTL_3B_2	3B	wPt-664724–P39/M31-2	92.92	2.65	GY, HI, LY, NG, PH, SL, SN, SNS, TKW, TN
MQTL_3B_3	3B	P39/M50-2–cfb3059	109.1	2.07	75%G, SNS, TKW
MQTL_3B_4	3B	barc176–wmc632	114.03	2.38	75%G, GY, NG, SNS, TKW, TMRS
MQTL_3B_5	3B	cgt/ctcg-146–wPt-666764	119.72	0.38	75%G, DH, GY, SL, SNS, TKW, TMRS
MQTL_3D_1	3D	cfd223–wPt-743340	32.58	9.44	GZnC, SN, TKW
MQTL_3D_2	3D	Xgwm892–wPt-8914	66.11	14.3	PH
MQTL_3D_3	3D	wPt-733972–wPt-666681	121.94	3.88	BM, GCuC, GFeC, GPC, GY, HI, PH
MQTL_3D_4	3D	wPt-664771–wPt-742685	140.91	13.53	BM, GFeC, GPC, GY, HI, PH
MQTL_3D_5	3D	wPt-741976–wPt-740544	218.00	9.27	GSeC, GZnC
MQTL_4A_1	4A	wPt-664971–BE399880	54.58	1.71	GL, GPC, GPL, GY, GZnC, NG, SNS
MQTL_4A_2	4A	tgc/agc-166–cfd30	73.07	1.94	DH, GL, GPL, GY, GZnC, HW, NG, SDS, SN, SNS, TKW
MQTL_4A_3	4A	wPt-3374–wmc0258	88.37	0.3	FWA, GL, GPC, GPL, HW, MDR, NG, SDS, TKW
MQTL_4A_4	4A	wmc776–wPt-9305	105.92	1.62	FWA, GCuC, GL, GPL, HW, LDMA, NG, SDS, SNS, TKW
MQTL_4A_5	4A	Xgwm832–Xmag3733	135.87	0.71	FWA, GCuC
MQTL_4B_1	4B	wPt-0037–wmc0047	9.07	4.66	DA, DPM, HW, MTI, PH, SD, SDS
MQTL_4B_2	4B	wPt-3608–wmc125	11.84	3.76	DA, DPM, HW, MTI, PH, SD, SDS
MQTL_4B_3	4B	gwm0149–Xcfd222	19.71	7.9	GN, GY, HW, KW, MTI, PH, SD, SDS, TKW
MQTL_4B_4	4B	wmc710–Xbarc1096	28.20	0.01	GN, HW, KW, MTI, PDMA, PH, SD, SDS, TKW
MQTL_4D_1	4D	Rht-D1–wmc285	17.98	1.87	GCuC, GFeC, GMnC, GY, SN, SNS
MQTL_4D_2	4D	wPt-732586–Xsrap11a	41.00	2.06	GFeC, GMnC, GZnC, SN
MQTL_4D_3	4D	cfd65–gwm609	100.02	5.4	GFeC, GMnC, GSeC, SD
MQTL_4D_4	4D	barc108–Xbarc1183	172.97	6.6	GFeC, GZnC
MQTL_5A_1	5A	wPt-6048–barc10	1.87	4.02	DA, DPM, PT
MQTL_5A_2	5A	Xcwem32.2–wmc59	46.08	5.03	DPM, LDMA
MQTL_5A_3	5A	Xbarc358.2–barc40	69.46	3.35	75%G, DA, DPM, GFD, MRS, PH, SHS, SW
MQTL_5A_4	5A	wPt-9834–gwm126	78.56	3.22	75%G, DA, DPM, GFeC, GWe, GY, LS, LY, MRS, SHS
MQTL_5A_5	5A	gwm595–Xbarc247	88.51	5.49	GFeC, GZnC
MQTL_5B_1	5B	cfd5–BE404594-175	0.00	1.65	DPM
MQTL_5B_2	5B	BE404594-175–wmc773	2.20	1.07	GY
MQTL_5B_3	5B	wPt-6135–gwm540	16.10	4.55	DH, GY, SL, TKW
MQTL_5B_4	5B	gdm116–gwm271	40.43	0.28	DH, GFD, GY, LDMA
MQTL_5D_1	5D	Xgdm99.2–Xbarc286	40.43	4.17	25%G, KL, PGMS, SNS
MQTL_5D_2	5D	cfa2104–ww152	47.41	0.34	25%G, DST, GPC, HW, GY, KH, KL, NG, PGMS, SN, WGC
MQTL_6A_1	6A	wPt-1381–wPt-0938	16.14	5.04	GWe, PH, TKW
MQTL_6A_2	6A	agg/cat-6–wPt-2636	26.8	2.68	NG, PDMA, SNS
MQTL_6A_3	6A	Xgwm82–wmc807	58.75	5.2	BM, NG, SNS
MQTL_6A_4	6A	Xgwm732–Xswes123.3	72.28	2.11	SNS
MQTL_6A_5	6A	wmc206–cwem49f	108.86	3.32	SNS, TKW
MQTL_6B_1	6B	gctg/ctt-1–agc/tgc-3	48.00	7	GY, MDR
MQTL_6B_2	6B	Dupw216–aca/ctga-7	77.00	5.1	DH
MQTL_6B_3	6B	act/gcg-11–agc/tgc-7	93.87	2.71	SHS, SW
MQTL_6B_4	6B	wPt-7662–gwm613	120.97	4.47	DA, PH
MQTL_6B_5	6B	wmc486–wmc487	130.30	5.1	PGMS, PH, TKW
MQTL_6B_6	6B	wPt-2786–barc0045	138.66	4.87	GY, PGMS, TKW
MQTL_6B_7	6B	cfa2110–agc/ctc-6	159.43	5.9	GY, PGMS, TKW, TMRS
MQTL_6B_8	6B	barc0247–wPt-1325	184.32	15.62	NG, PGMS
MQTL_6D_1	6D	cfd0049–Xswes123.6	8.23	19.83	PT, SN
MQTL_6D_2	6D	Xswes123.7–Xcft3103	43.93	15.84	GY, SN
MQTL_6D_3	6D	wmc749–barc175	65.91	6.6	GY, NG, SN
MQTL_6D_4	6D	Xcfa2114–gpw95010	85.38	21.45	GY, SN
MQTL_7A_1	7A	wmc497–wPt-6217	45.93	1.82	CID, DA, DGC, DPM, GFD, GFeC, GPC, GZnC, KH, NG, PH, PT, SDS, SL, SNS, TKW
MQTL_7A_2	7A	cfd13–gwm4	51.07	1.48	CID, DA, DGC, DPM, GFD, GFeC, GPC, GZnC, KH, LS, NG, PH, PT, SDS, SHS, SNS, TKW, TMRS
MQTL_7A_3	7A	Xwmc475.1–cfa2257	59.28	0.75	CID, DA, DGC, DPM, GFD, GFeC, GPC, GY, GZnC, KH, NG, PH, PT, SDS, SHS, SL, SNS, TKW
MQTL_7A_4	7A	wPt-1259–Xmag2931.3	74.58	3.1	DGC, GPC, KH, PH, SDS, TKW
MQTL_7B_1	7B	U260–gwm569	61.32	4.04	50%G, DH, DPM, GFeC, GL/GW, GPL, NG, SL
MQTL_7B_2	7B	wPt-4342–wPt-7813	72.66	0.89	50%G, DH, DPM, GFD, GFeC, GFR, GL/GW, GPL, GY, GZnC, KH, NG, PH, SD, SL, TKW, TMRS
MQTL_7B_3	7B	wPt-6372–barc176	83.18	2.37	50%G, DH, DPM, GCuC, GFeC, GFR, GL/GW, GPL, GY, KH, NG, PH, SL, TKW
MQTL_7B_4	7B	Xcau12.3–barc126	98.82	8.22	50%G, DPM, GFeC, GL/GW, GPL, GY, KH, PH, TKW
MQTL_7B_5	7B	Xbarc1073.2–wmc10	121.38	3.87	GFeC, GL/GW, GPL, KH, NG, SN, SNS, TKW
MQTL_7B_6	7B	Xgwm3036–gwm146	132.26	2.72	GFeC, GL/GW, GPL, NG, SNS, TKW
MQTL_7D_1	7D	wmc121–wmc489	89.41	4.94	50%G, SD, TMRS
MQTL_7D_2	7D	wmc473–wmc94	98.52	5.16	GY, SD
MQTL_7D_3	7D	gtg/cagt-4–wmc824	121.60	3.82	LY, PH
MQTL_7D_4	7D	barc53–cfd0083	149.45	35.94	SHS

* *The full name of assessed traits are displayed in Table 1*.

### Functional Identification of Candidate Genes

The genomic positions of the stable MQTLs and the number of functional candidate genes (CGs) in their intervals are reported in Table 3. The range for the number of the CGs annotated in the tested meta-QTLs was between 20 and 802. The MQTL_4D_1, MQTL_4B_3, MQTL_3B_1, and MQTL_5A_4 harbored the greatest number of CGs. Among the detected CGs on MQTL regions, several well-known genes including *psbL* (21.896318-21.896434 Mb), *psbT* (21.905522-21.905638 Mb), *rpl33* (21.899696-21.899896 Mb), and *rps4* (24.160684-24.161289 Mb) genes were located in the MQTL_3D_4 region. In addition, the *miR166* gene was detected in the MQTL_4D_1 (450.196302-450.196403 Mb) and MQTL_7A_4 (561.152174-561.152339 Mb) regions. Furthermore, several CGs with unknown annotation in wheat and were orthologous to genes in rice were identified (Supplementary Tables 4, 5). Overall, some CGs, such as *TraesCS2A02G141400, TraesCS3B02G040900, TraesCS4D02G323700, TraesCS3B02G077100*, and *TraesCS4D02G290900* were uncovered with a possible role in micronutrient contents, yield, and yield-related traits.

**Table 3:** The genomic position of most stable meta-quantitative trait loci (MQTLs) on the wheat genome and number of genes in their genomic intervals.

**MQTL**	**Chr. No**	**Genomic position on the wheat genome (Mb^¶^)**	**Number of initial trait QTLs**	**Number of genes laying at the MQTL interval**	**MQTL**	**Chr. No**	**Genomic position on the wheat genome (Mb^¶^)**	**Number of initial trait QTLs**	**Number of genes laying at the MQTL interval**
MQTL_1A_4	1A	508.04–511.15	8	48	MQTL_4B_1	4B	601.95–640.98	7	348
MQTL_1B_1	1B	16.23–27.30	7	170	MQTL_4B_2	4B	644.87–652.88	7	113
MQTL_1B_2	1B	564.78–571.06	9	53	MQTL_4B_3	4B	509.04–576.50	9	479
MQTL_1B_3	1B	678.45–681.00	7	30	MQTL_4B_4	4B	597.03–608.25	9	105
MQTL_2A_1	2A	77.88–91.56	10	160	MQTL_4D_1	4D	425.23–490.12	6	802
MQTL_2A_2	2A	38.75–56.93	12	189	MQTL_5A_3	5A	439.58–444.92	8	46
MQTL_2A_3	2A	504.28–507.77	11	23	MQTL_5A_4	5A	671.39–702.96	10	427
MQTL_2B_1	2B	686.82–707.65	8	223	MQTL_5D_2	5D	28.96–34.08	11	38
MQTL_2B_2	2B	760.14–762.08	7	28	MQTL_7A_1	7A	1.47–4.99	16	86
MQTL_3B_1	3B	16.67–50.54	18	457	MQTL_7A_2	7A	49.58–53.07	18	30
MQTL_3B_2	3B	784.63–788.79	11	58	MQTL_7A_3	7A	25.06–41.48	18	233
MQTL_3B_4	3B	822.58–830.11	6	112	MQTL_7A_4	7A	560.02–563.50	6	35
MQTL_3B_5	3B	814.18–826.25	7	221	MQTL_7B_1	7B	32.55–36.92	9	55
MQTl_3D_3	3D	31.87–38.29	7	63	MQTL_7B_2	7B	669.71–693.34	17	241
MQTl_3D_4	3D	16.88–30.37	6	250	MQTL_7B_3	7B	557.05–582.70	14	170
MQTL_4A_1	4A	474.79–488.25	7	66	MQTL_7B_4	7B	38.87–41.30	9	20
MQTL_4A_2	4A	151.24–182.24	11	116	MQTL_7B_5	7B	741.47–744.92	8	107
MQTL_4A_3	4A	632.62–656.77	9	204	MQTL_7B_6	7B	729.40–740.72	6	93
MQTL_4A_4	4A	732.61–734.94	10	59	–	–	–	–	–

¶ *Mb, represents mega base pair*.

### Traits Analysis Within MQTLs

**Figure 6:**
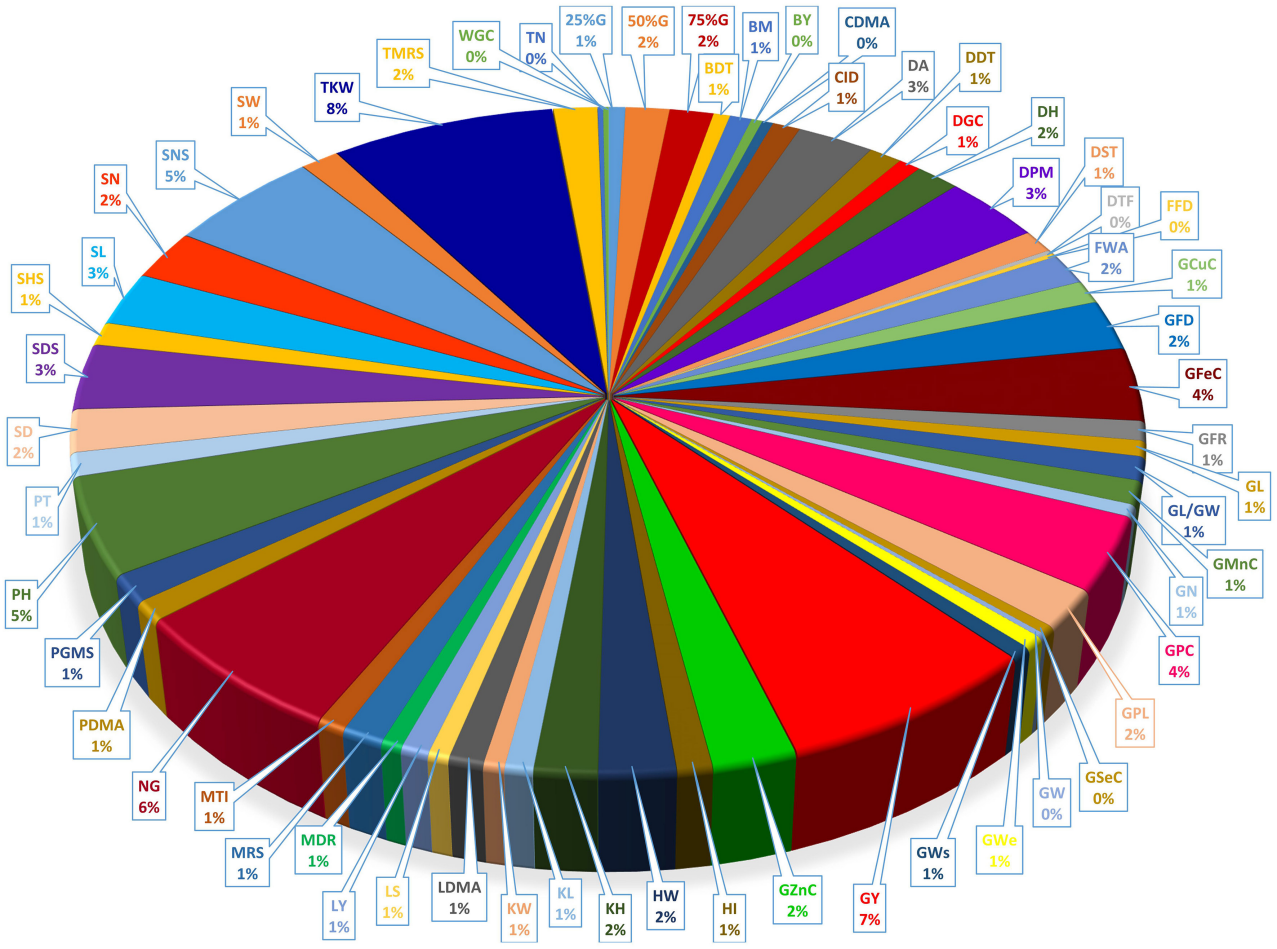
Distribution of QTLs controlling different traits in detected MQTL regions.

Our data show the unequal contribution of individual QTL across the detected MQTLs (Figure 6 and Supplementary Table 3). Individual QTL for TKW were present in 41 of the 100 MQTL regions, the most for any agronomic trait, followed by GY (38 MQTL). Among quality traits, QTLs for GPC was the most distributed QTLs which were located among 19 MQTLs. The GFeC and GZnC QTLs were presented in 19 and 12 MQTLs, respectively (Supplementary Table 3).

Analysis for the co-localization of QTLs revealed that QTLs for GY and TKW were frequently co-localized with QTLs of the target traits (GPC, GFeC, and GZnC). The QTLs for TKW showed 52% co-localizations with QTLs for GPC (Supplementary Table 6). The GY QTLs showed 52% co-localization with the QTLs for GFeC. Results also indicated 66% co-localization between grain Fe and grain Zn QTLs. Co-localization frequency for GY (*R*^2^ = 80.81%) was strongly associated with the total number of MQTL for a trait. The association of co-localization frequency with the overall number of MQTL for a trait was relatively strong for GPC (R^2^ = 65.58%), GFeC (R^2^ = 58.74%), and GZnC (R^2^ = 56.00%). For most traits, association with target traits (GPC, GZnC, GFeC, and GY) did not differ from the expected on the basis of chi-squared analysis. However, the association of traits with GY, GPC, GFeC, and GZnC as target traits was more than expected (Supplementary Table 6).

### Comparison of the Identified MQTLs and QTL Mapping in the Wheat GWAS

The comparison of the MQTL locations with genome-wide association studies (GWAS) QTL regions showed that 21 significant signals (SNPs-linked QTLs) of the available wheat GWAS map were co-located with MQTLs of seven of the 35 traits tested in our study (Table 4). The results indicated the co-localization of significant single-nucleotide polymorphisms (SNPs) for GY (6 SNPs), a number of grains per spike (NG) (1 SNPs), plant height (PH) (4 SNPs), spike length (SL) (2 SNPs), spike number (SN) (1 SNPs), spike number per spike (SNS) (4 SNPs), and thousand kernel weight (TKW) (3 SNPs) traits in the wheat GWAS with the identified MQTLs in our study. For instance, the MQTL_4A_3, MQTL_4A_4, and MQTL_4B_3 identified for TKW in our study were positioned in the genomic regions of the major signal reported for TKW in the wheat GWSA map. Overall, the co-located MQTLs and significant GWAS signals were distributed on chromosomes 1B, 2B, 3B, 4A, 4B, 4D, 5A, 7A, and 7B (Table 4).

**Table 4:** The collinear meta-quantitative trait loci (MQTLs) with the significant loci in wheat genome-wide association studies.

Trait^*^	**Wheat MQTL**	**Chromosome (genomic position in Mb)**	**SNP marker name (genomic position in Mb)**	**Wheat GWAS references**
GY	MQTL_3B_1	3B (16.67–50.54 Mb)	AX_109881378 (20.5–22.0)	Li et al., 2019
	MQTL_4A_2	4A (151.24–182.24 Mb)	M8680 (157.56)	Mathew et al., 2019
	MQTL_5A_4	5A (671.39–702.96 Mb)	AX-110458478 (692.17)	Hu et al., 2020
			AX-108839508 (692.16)	
			AX-109388349 (692.18)	
			AX-108829895 (692.39)	
NG	MQTL_7B_2	7B (669.71–693.34 Mb)	S7B_687521301 (687.52)	Jamil et al., 2019
PH	MQTL_4D_1	4D (425.23–490.12 Mb)	AX-95235641 (442.17)	Hu et al., 2020
	MQTL_7B_2	7B (669.71–693.34 Mb)	AX-110149206 (676.25)	Hu et al., 2020
			AX-95658823 (675.28)	
			AX-95149761 (680.08)	
SL	MQTL_1B_2	1B (564.78–571.06 Mb)	AX-109901032 (566.19)	Li Q. et al., 2020
	MQTL_7A_3	7A (25.06–41.48 Mb)	AX-109394807 (29.32)	Hu et al., 2020
SN	MQTL_4A_2	4A (151.24–182.24 Mb)	AX-109066809 (179.94)	Li Q. et al., 2020
SNS	MQTL_2B_1	2B (686.82–707.65 Mb)	AX-111551006 (706.93)	Li Q. et al., 2020
	MQTL_4D_1	4D (425.23–490.12 Mb)	AX-169338181 (433.00)	
			AX-111020167 (471.23)	
	MQTL_7A_1	7A (1.47–4.99 Mb)	AX-111542213 (4.55)	
TKW	MQTL_4A_4	4A (732.61–734.94 Mb)	S4A_733664972 (733.66)	Jamil et al., 2019
	MQTL_4A_3	4A (632.62–656.77 Mb)	AX-111600193 (642.37)	Li Q. et al., 2020
	MQTL_4B_3	4B (509.04–576.50 Mb)	AX-94402252 (564.39)	Hu et al., 2020

* *Full names of traits are displayed in Table 1*.

### Orthologous MQTL Mining of Wheat in Rice and Maize

**Figure 7:**
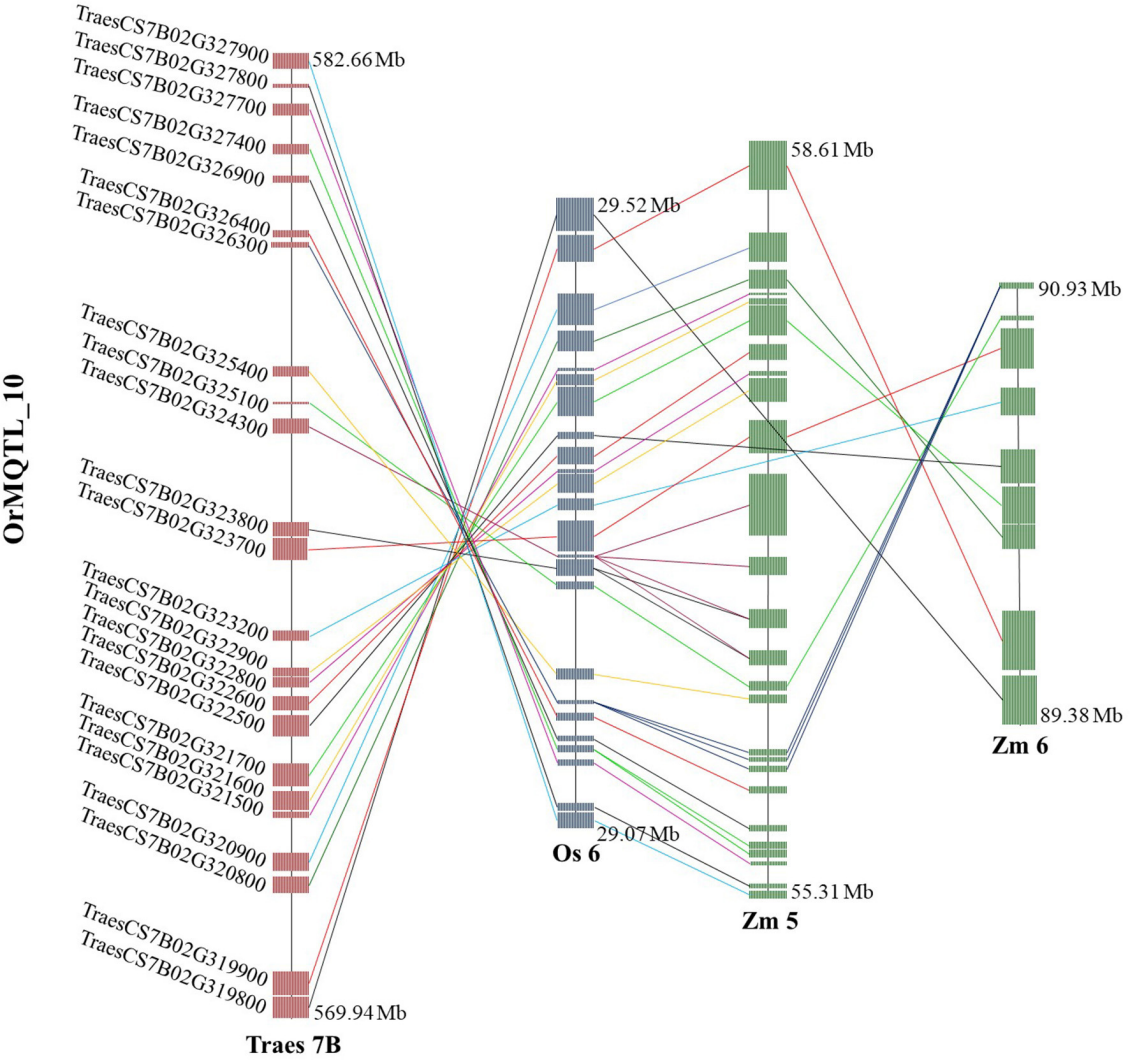
The syntenic region of meta-quantitative trait loci (MQTLs) among the wheat, rice, and maize. Orthologous MQTL (OrMQTL)_10 indicates syntenic regions among identified MQTLs for grain yield (GY) control in wheat (MQTL_7B_3), rice (MQTL-YLD14), and maize (MQTL5.5 and MQTL66). The genomic position, chromosome number, and common genes among the wheat, rice, and maize are indicated.

The comparative analysis for QTLs in wheat, rice, and maize resulted in the identification of orthologous MQTL (OrMQTL). Nine OrMQTLs were detected for wheat and rice including five OrMQTL for GY and two for PH, and GFeC/GZnC, respectively. Moreover, seven OrMQTL were identified in wheat and maize consisting of six and one OrMQTL for GY and PH, respectively. Among the uncovered OrMQTLs, the OrMQTL_10 was a cross-species QTL in wheat, rice, and maize (Figure 7; Table 5). The MQTL_7B_2, MQTL_3B_4, MQTL_4D_1, and MQTL_5A_4 for GY in wheat were in the co-linear regions for rice GY MQTLs on chromosome 6 (MQTL6-2), 1 (MQTL-YLD3), 3 (MQTL-YLD9), and 11 (MQTL-YLD19), respectively (Table 5). The wheat MQTL_4B_1 and MQTL_7B_5 were in the co-linear region of MQTLs for PH on chromosome 3 (MQTL-PH11) and 10 (MQTL-PH26) in rice (Table 5). In addition, wheat MQTL_2A_1, MQTL_4D_1 and MQTL_4D_1 were in the co-linear regions of MQTLs of GFeC and GZnC on chromosome 7 (rMQTL7.1), 6 (rMQTL6.3) and 7 (rMQTL7.2) in rice (Table 5). The MQTL_1B_1, MQTL_2A_1, MQTL_4A_3, MQTL_4B_2, and MQTL_4B_3 on wheat chromosomes 1B, 2A, 4A, and 4B were in located in the co-linear regions of the MQTLs of GY on chromosomes 1 (MQTL7), 5 (MQTL29), 1 (MQTL10), 8 (MQTL44), and 2 (MQTL23) in maize (Table 5). Furthermore, an OrMQTL for MQTL_3D_3 on wheat chromosome 3D was detected on chromosome 10 (MQTL107) harboring the MQTL for PH in maize (Table 5). One of the wheat MQTLs (MQTL_7B_3) on chromosome 7B was located in the syntenic region of maize MQTLs chromosome 5 (MQTL5.5) and 6 (MQTL66), respectively, as well as rice MQTLs on chromosome 6 (MQTL-YLD14) for GY (Figure 7; Table 5). The genes located at the OrMQTL regions and their annotations were reported in Supplementary Table 7. Interestingly, the well-known and proved orthologous genes including *OsCYP72A18, OsFbox146, NAC22, SD37, BRD2, OsMAPK4*, and *ZmMPK5* were identified in rice and maize OrMQTLs.

**Table 5:** The OrMQTLs detected in rice and maize according to the syntenic region with MQTLs in wheat.

**Trait**	**Orthologous MQTL (OrMQTL)**	**Wheat MQTL**	**Wheat chr. no. (genomic position in Mb)**	**Rice/Maize original MQTL name**	**Rice chr. no. (genomic position in Mb)**	**Maize chr. no. (genomic position in Mb)**	**Rice/Maize MQTL reference**
GY	OrMQTL_1	MQTL_7B_2	7B (674.1981–674.2014)	MQTL6-2	6 (3.1029–3.1075)	–	Lei et al., 2018
	OrMQTL_2	MQTL_3B_4	3B (828.4199–828.7654)	MQTL-YLD3	1 (25.0457–25.0494)	–	Khahani et al., 2020
	OrMQTL_3	MQTL_4D_1	4D (459.6856–459.6870)	MQTL-YLD9	3 (14.6804–14.6827)	–	Khahani et al., 2020
	OrMQTL_4	MQTL_5A_4	5A (700.1772–700.1789)	MQTL-YLD19	11 (10.4749–10.4777)	–	Khahani et al., 2020
	OrMQTL_5	MQTL_1B_1	1B (27.1500–27.1501)	MQTL7	–	1 (224.7216–224.7217)	Wang et al., 2013
	OrMQTL_6	MQTL_2A_1	2A (82.1848–82.1880)	MQTL29	–	5 (19.2226–19.2254)	Wang et al., 2013
	OrMQTL_7	MQTL_4A_3	4A (647.0874–647.0936)	MQTL10	–	1 (275.0211–275.0279)	Wang et al., 2013
	OrMQTL_8	MQTL_4B_2	4B (649.4698–649.4752)	MQTL44	–	8 (47.1438–47.1520)	Wang et al., 2013
	OrMQTL_9	MQTL_4B_3	4B (518.0952–518.0999)	MQTL23	–	2 (122.6755–122.6792)	Wang et al., 2016
	OrMQTL_10	MQTL_7B_3	7B (569.4388–582.6560)	MQTL-YLD14	6 (28.8459–29.5240)	–	Khahani et al., 2020
			7B (568.6510–582.6560)	MQTL5.5	–	5 (55.3131–58.7190)	Semagn et al., 2013
			7B (568.1069–579.8265)	MQTL66	–	6 (89.3129–90.9312)	Wang et al., 2016
PH	OrMQTL_11	MQTL_4B_1	4B (619.5886–635.8693)	MQTL-PH11	3 (1.8624–2.2252)	–	Khahani et al., 2020
	OrMQTL_12	MQTL_7B_5	7B (741.5702–741.5720)	MQTL-PH26	10 (13.3596–13.3632)	–	Khahani et al., 2020
	OrMQTL_13	MQTL_3D_3	3D (33.2949–33.2960)	MQTL107	–	10 (69.4836–69.4849)	Wang et al., 2016
GFeC, GZnC	OrMQTL_14	MQTL_2A_1	2A (88.2949–88.2975)	rMQTL7.1	7 (7.3945–7.3976)	–	Raza et al., 2019
	OrMQTL_15	MQTL_4D_1	4D (484.6841–484.6880)	rMQTL6.3	6 (21.3970–21.3993)	–	Raza et al., 2019
		MQTL_4D_1	4D (461.4899–461.4930)	rMQTL7.2	7 (19.9760–20.0812)	–	Raza et al., 2019

*OrMQTL, orthologous MQTL; GY, grain yield; PH, plant height; GFeC, grain Fe content; GZnC, grain Zn content*.

## Discussion

### Quantitative Trait Loci and Meta-Quantitative Trait Loci Distribution Over the Wheat Genome

Analysis of the genetic control of quantitative traits is a challenge in plant breeding that is due to the complexity and the difficulty of stacking numerous alleles for their control (Izquierdo et al., 2018). Meta-quantitative trait loci (MQTL) analysis made possible the consolidation of individual QTL into MQTL regions. The MQTL analysis and the comparative genomic approaches help to identify consensus QTLs and conserved genes across genomes and species. In the current MQTL analysis, we used information from over 60% of the individual QTLs to identify MQTLs that showed the statistical power of MQTL analysis for combining wheat QTLs that were in the range of 54–84% that have been reported in the previous studies of MQTL in wheat (Löffler et al., 2009; Acuña-Galindo et al., 2015; Soriano and Alvaro, 2019). In this study, we narrowed down the confidence interval (CI) of the detected MQTL regions compared to the initial QTLs used in our MQTL analysis. The efficacy of MQTL analysis in refining the CI for previously known QTLs as well as for validating their effects across different genetic backgrounds and environments is well-demonstrated (Goffinet and Gerber, 2000; Wu and Hu, 2012).

The results showed that QTLs and MQTLs had higher density in the non-telomeric and near-centromeric regions. The QTL result from the genetic segregation of sequence polymorphisms at functional elements, such as regulatory sequences upstream of genes and/or coding sequences (Flint and Mackay, 2009; Salvi and Tuberosa, 2015). Therefore, it is expected that QTL density on a genetic map is driven by gene density, polymorphism rate at functional sites in genetic regions, and the frequency of recombination. This finding was in line with the result of Martinez et al. (2016), which illustrated higher QTLs densely mapped to the near centromeres on the genetic maps.

In the quality traits category, QTLs for grain protein content (GPC), grain Fe content (GFeC), and grain Zn content (GZnC) traits were the most observed QTLs in the identified MQTLs (Supplementary Table 2). In agronomic traits, QTLs for thousand kernel weight (TKW), grain yield (GY), and number of grains per spike (NG) were the most frequent QTLs identified in the MQTLs regions. The outcome of a MQTL analysis in tetraploid wheat revealed that more than 10 loci were associated with TKW (Avni et al., 2018). Besides, in another MQTL analysis in wheat, individual QTLs for TKW, GY, and kernel number had the highest number of individual QTLs in MQTL regions. This result shows the importance of these QTLs for tested traits. The probable assumptions for a higher frequency of QTLs for agronomic traits are easy to measure and frequent data for these traits in different genetic mapping studies. On the other hand, the TKW, GY, NG, GPC, GFeC, and GZnC traits are multi-genic, highly heritable, and relatively insensitive to the environment (especially, TKW) which suggests a high likelihood of different populations carrying different suites of relevant alleles (Cooper et al., 1995; Bezant et al., 1997; Avni et al., 2018; Velu et al., 2018; Zhang et al., 2020).

### MQTLs and Functional Candidate Genes

Gene annotation analysis for the MQTL regions helps clarify our understanding of their genetic architecture and refining the targets of breeding for these traits. The results of functional genomics for the identified MQTLs showed that several well-known genes consisted of *psbL* for electron transfer in photosystem II (PSII) (Ozawa et al., 1997), *psbT* encoding a PSII subunit for maintaining optimal PSII activity under adverse growth conditions (Monod et al., 1994), *rpl33*f or structural constituent of ribosome and translation which confers tolerance to cold stress (Rogalski et al., 2008; Moin et al., 2017), and the *rps4* gene for the regulation of translational fidelity in wheat were located in the MQTl_3D_4 region. In addition, the *miR166* gene was detected in the MQTL_4D_1 and MQTL_7A_4 regions. Comparative genomic approaches start with making some form of alignment of genome sequences and looking for orthologous sequences in the aligned genomes and checking to what extent those sequences are conserved. Based on these, genome and molecular evolution are inferred and this may, in turn, be put in the context of phenotypic evolution or population genetics. Analysis of the conservation and diversification of the *miR166* family have shown that *miR166* members play a wide and important regulatory role in seed development. More recently, a short-tandem target mimic (STTM) method was used to verify if *miR166* regulates important agronomic traits in rice (Zhang et al., 2017). In a study, transgenic STTM165/166 plants showed significantly reduced seed number and sterile siliques in Arabidopsis, suggesting that *miR166* plays a vital role in seed development and it might be useful evidence to improve inferior grain size in wheat (Wang et al., 2018).

Among the detected and annotated genes in the MQTLs regions in this study, the *OsMED9* (*TraesCS3B02G077100*), which is an orthologous gene of rice, was identified in the MQTL_3B_1 region that was associated with GY and its components. A diverse array of MED genes has been identified for the regulation of GY and yield components in crop plants (Malik et al., 2016). The results indicated that the MQTL_4D_1 of our study was located in the regions of the rice orthologous *BG1* (*TraesCS4D02G290900*), *OsIDD1* (*TraesCS4D02G262500*), and *OsPAO* (*TraesCS4D02G309000*) genes in wheat. These genes are associated with metal ion binding, flowering time, days to heading, senescence, gravitropism, yield, and yield-related traits (Wu et al., 2008; Liu et al., 2015; Chen et al., 2016; Deng et al., 2017; Mishra et al., 2017). Overexpression of *BG1* leads to larger grain size in rice (Liu et al., 2015) and manipulation of *BG1* increases plant biomass, grain size, and GY in rice and Arabidopsis (Liu et al., 2015; Mishra et al., 2017). *OsIDD1* could rescue the never-flowering phenotype of *rid1* by a transition from vegetative to reproductive growth in rice (Deng et al., 2017), and the *PAOs* protein-encoding genes (Chen et al., 2016) regulate cellular polyamine levels which are critical for embryogenesis (Bertoldi et al., 2004; De-la-Pena et al., 2008), germination (Bethke et al., 2004; Liszkay et al., 2004), root growth (Cona et al., 2005), flowering and senescence (Kakkar and Sawhney, 2002), and mineral deficiency (Moschou et al., 2008, 2009). The orthologous *Ghd7* (*TraesCS5A02G541200*) that was among 427 genes located in the MQTL_5A_4 region in our study involves photoperiodism, flowering, days to heading, plant height (PH), and yield traits. Enhanced expression of *Ghd7* under long-day conditions delays heading and increases PH and panicle size in rice. The *Ghd7* gene plays crucial role in increasing the productivity and adaptability of rice globally (Xue et al., 2008). The uncovered rice orthologous *D27* (*TraesCS7B02G319100*) and *BRD2* (*TraesCS7B02G484200*) genes in wheat in chromosome 7B region are known to control tillering, heading date, PH, and yield traits in rice (Hong et al., 2005; Lin et al., 2009; Liu et al., 2016).

The identified rice orthologous *D18* (*TraesCS2B02G570800*), *OsRPK1* (*TraesCS5D02G034200*), *DRUS1*, and *DRUS2* (*TraesCS4A02G133800*) genes within the MQTL_2B_2, MQTL_4A_2, and MQTL_5D_2 regions of our MQTLs were associated with PH in wheat (Itoh et al., 2002; Zou et al., 2014; Pu et al., 2017). Manipulating these genes lead to varieties with dwarf and semi-dwarf phenotype and such phenotypes possess short and strong stalks, exhibit less lodging, and a greater proportion of assimilation partitioned into the grain, resulting in further yield increases (Hedden, 2003).

The identified rice orthologous *OsRLCK189* (*TraesCS1A02G317300*), *OsGH3-4* (*TraesCS1A02G320200*), *FT-L* (TraesCS2B02G511400), *OsRLCK57* (*TraesCS3B02G600300*), *OsHK1* (*TraesCS2B02G495500*), *AIM1* (*TraesCS3D02G077200*), and *WOX11* (*TraesCS2A02G100700*) genes in wheat was located in MQTL_1A_4, MQTL_2A_2, MQTL_2B_1, MQTL_3B_5, and MQTl_3D_3 regions show a role in root growth and inflorescence and floral development (Richmond and Bleecker, 1999; Izawa et al., 2002; Vij et al., 2008; Ogiso-Tanaka et al., 2013; Zhao et al., 2015, 2020; Cheng et al., 2016; Xu et al., 2017; Lehner et al., 2018; Kong et al., 2019). Inflorescence development in cereals directly affects grain number and size which are key determinants of yield (Yamburenko et al., 2017).

The rice orthologous *OsMTP12* (*TraesCS2A02G141400*), *OsMTP9* (*TraesCS3B02G040900*), and *OsMTP1* (*TraesCS4D02G323700*) belonging to the metal tolerance protein (MTP) gene family were identified in the MQTL_2A_1, MQTL_3B_1, and MQTL_4D_1 interval of the wheat genome in the present study. The MTP gene family plays a critical role in metal transport, mainly in Zn, Mn, Fe, Cd, Co, and Ni, metal homeostasis, and tolerance (Gustin et al., 2011; Zhang and Liu, 2017; Ram et al., 2019). The increased expression of MTP genes during seed development and their potential role in metal homeostasis during the seed filling stage had been documented (Ram et al., 2019). The *MTP1* and *MTP12* genes share a characteristic histidine-rich loop toward the c-terminal, which is known to have a role in Zn-binding (Ram et al., 2019).

The MQTL_1A_4, MQTL_3B_5, MQTL_4B_1, MQTL_4B_2, MQTL_4B_3, MQTL_4B_4, MQTL_7A_1, and MQTL_7A_4 interval regions harbored rice orthologous *VAL1* (*TraesCS4B02G314600*), *PROG1* (*TraesCS4B02G354000*), *D14* (*TraesCS4B02G258200*), *LPL2* (*TraesCS4B02G308000*), *OsINV3* (*TraesCS7A02G009100*), and *OsRLCK218* (*TraesCS7A02G385300*) genes with a role in diverse development and growth traits including leaf development, PH, shoot branching, panicle and tiller number, grain number, grain and spikelet size, and GY (Vij et al., 2008; Zhou et al., 2016; Morey et al., 2018; Wu et al., 2018; Yao et al., 2018; Zhang et al., 2018; Deng et al., 2020). These candidate genes (CGs) at the detected MQTL regions can potentially have the same function as their orthologous varieties in rice and therefore regulate various developmental and growth-related traits in wheat. Identification of these confines stable chromosomal regions and CGs that influence economically important traits in wheat can help to expedite wheat improvement in future breeding programs.

The MQTLs detected in this study will help identify CGs in these regions responsible for desirable traits and generate allele-specific markers through allele mining (Leung et al., 2015; Ogawa et al., 2018) for marker-assisted selection (MAS) application in pre-breeding population. Allele mining is a promising approach to dissect naturally occurring allelic variation at QTLs/CGs controlling desirable traits (e.g., yield, quality, and micronutrient content) which has potential applications in crop improvement programs (Kumar et al., 2010; Gokidi et al., 2017; Kumari et al., 2018). The data raised from this MQTL study help to refine genomic targets for validation and subsequent development of haplotypic markers in breeding programs. Information of the identified MQTLs can also be used for genetic transformation or allele screening in germplasm collections (ecoTilling) for finding new alleles capable of improving yield, quality, and micronutrient traits (Izquierdo et al., 2018; Belzile et al., 2020). Additionally, a promising application of the variation within these MQTL regions might be their introduction as fixed effects in genomic selection (GS) models to increase the accuracy of the prediction models in their use in wheat breeding programs (Spindel et al., 2016; Izquierdo et al., 2018).

### MQTL and Traits Analysis

The QTLs projection on a consensus map allows for the inspection of co-location across traits and categories, which is especially relevant for complex traits (Delfino et al., 2019). The association between trait classes by analyzing the co-localization frequency of individual trait-QTLs demonstrated that TKW with 55% and 63% co-localization frequencies was frequently associated with GY and GPC. In addition, GY and GFeC had the highest co-localization frequency with GFeC (52%) and GZnC (66%), respectively. The results of MQTL analysis of our study confirmed correlations identified for QTLs of TKW and GY (An-Ming et al., 2011; Azadi et al., 2014; Mahdi-Nezhad et al., 2019), TKW and GPC (Wang L. I. et al., 2012; Goel et al., 2019), and GFeC and GZn (Roshanzamir et al., 2013; Pu et al., 2014; Tiwari et al., 2016; Liu et al., 2019) in other studies. Co-localization of QTLs for correlated traits has been identified by Wang et al. (2018). Co-localization of QTLs could be due to the pleiotropy or the presence of different linked genes in the same regions that can partly explain the correlation that exists between traits (Bhatta et al., 2018). The tightly linked genomic regions or pleiotropy can partly explain the correlation that exists between traits (Crespo-Herrera et al., 2016). The result of co-localization frequency of target traits (GY, GPC, GFeC, and GZnC) with the detected MQTLs in our study showed interacting MQTLs which affects the association of traits at the genomic level. Acuña-Galindo et al. (2015) demonstrated that TKW was most frequently associated with GY in MQTL regions, with 57% co-localization. The co-localization of TKW and GPC QTLs has been observed in the genetic analysis of wheat (Goel et al., 2019). The positive and highly significant associations of GPC and TKW traits have been reported in different wheat populations in other studies (Peleg et al., 2009; Badakhshan et al., 2013; Krishnappa et al., 2017). According to Liu et al. (2019), mineral nutrient concentrations and yield components have shown common QTLs which is in line with co-localized QTLs for grain Zn, Fe QTLs in the tetraploid and hexaploid wheat in other studies (Peleg et al., 2009; Xu et al., 2012; Crespo-Herrera et al., 2016; Krishnappa et al., 2017; Velu et al., 2017). In a recent QTL analysis study on rice, grain Fe and grain yield QTLs were found to be co-localized (Dixit et al., 2019). Crespo-Herrera et al. (2016) suggested the possibility of simultaneous breeding for GFeC and GZnC traits due to their co-localized QTLs. The current co-localization analysis within MQTLs regions indicates the possibility of simultaneous breeding of micronutrient content, grain quality, and GY by pyramiding the QTL regions through marker-assisted selection (MAS). Using -MAS, the specific regions can be transferred to the elite wheat genotypes to simultaneously increase the contents of various traits (Saini et al., 2020). Therefore, an attempt to pyramid QTLs responsible for GY, TKW, GPC, GZnC, and GFeC may accelerate progress in wheat variety development. The QTL pyramiding strategy has been used for simultaneous improvement of traits through MAS in wheat (Wang P. et al., 2012; Feng et al., 2018; Gautam et al., 2020; Muthu et al., 2020). A clear understanding of the co-location of QTLs and their effect on target traits, such as grain Fe and Zn concentrations and yield is very important for using the major effect of QTLs in marker-assisted breeding (Swamy et al., 2018). The results of MQTL analysis in our study showed that the location of 18 MQTLs was in agreement with the position of the QTLs in the meta-QTL studies by Zhang A. et al. (2010), Acuña-Galindo et al. (2015), and Kumar et al. (2020). However, our work adds to this body of genomic mapping research by identifying new MQTL specific for GY, grain quality, and micronutrient content.

### Comparative Genomic Analysis and Orthologous MQTL

The genome-wide association studies (GWAS) approach can help in gene discovery and in the analysis of the genetic basis of complex traits for the improvement of wheat. Comparative analysis of the wheat GWAS with the identified current MQTLs suggested the co-localization of 12 MQTLs (MQTL_1B_2, MQTL_2B_1, MQTL_3B_1, MQTL_4A_2, MQTL_4A_3, MQTL_4A_4, MQTL_4B_3, MQTL_4D_1, MQTL_5A_4, MQTL_7A_1, MQTL_7A_3, and MQTL_7B_2) and significant genomic regions of wheat traits that have been identified in the wheat GWAS studies (Jamil et al., 2019; Li et al., 2019; Mathew et al., 2019; Hu et al., 2020; Li X. et al., 2020). Some of the QTL hotspots suggested common genetic markers for wheat traits for further use in marker-assisted breeding that increase genetic gain in breeding programs. The identified OrMQTLs in this study can facilitate the detection of the underlying regulatory genes with evolutionary history and conservative function. The current MQTL analysis defines a genome-wide landscape on the most stable genetic markers and CGs related to micronutrient content, yield, and yield-related traits as the most economically important traits in wheat. The straight-up comparative genomics in our study indicated high synteny between wheat, rice, and maize QTLs especially for GY suggesting possible corroborating evidence of acting the same way in different species that was in line with the results of other MQTL studies (Ahn et al., 1993; Moore et al., 1995; Gale and Devos, 1998; Feuillet and Keller, 1999; Minx et al., 2005). Despite the high interest in the identification of genes involved in GY and yield-related traits in maize and wheat as two economically important crops, the responsible genes have largely remained unknown due to their complex genomes. Given a close evolutionary relation among grass genomes (Gaut, 2002), a synteny analysis of wheat, maize, and rice through the identification of OrMQTLs enabled us to broaden our genetic information and leads to uncover the possible function of unknown CGs among these species. Here we selected the most promising wheat MQTLs to explore their conserved syntenic regions reported in similar MQTLs studies on the same traits in rice and maize to identify OrMQTLs (Table 5). For the wheat MQTL_3B_4 of our study, there is an MQTL in the syntenic region in rice (Khahani et al., 2020) controlling OrMQTL_2 containing a rice *OsCYP72A18* orthologous gene (TraesCS3B02G609400 and TraesCS3B02G609600) for grGY (Swamy and Sarla, 2011). Moreover, the wheat MQTL_4D_1 and MQTL_7B_2 of our study were located in the syntenic regions of the rice genome on chromosomes 3 (OrMQTL_3) and 6 (OrMQTL_1), respectively. These two OrMQTLs encompass *OsFbox146* (TraesCS4D02G288900) and *OsFbox297* (TraesCS7B02G405900) orthologous genes for genetic control of GY in rice (Swamy and Sarla, 2011; Lei et al., 2018). Furthermore, the syntenic region of wheat MQTL_4B_1 possessed the two orthologous rice genes, *NAC22* (TraesCS4B02G328600) and *CYP96B4/SD37* (TraesCS4B02G342400) located on chromosome 3 (OrMQTL_11) which control plant growth (Tamiru et al., 2015; Hong et al., 2016). In the syntenic regions of the MQTL_2A_1 and MQTL_4D_1 of our study, there was orthologous *OsZIP1/OsZIP8* (TraesCS2A02G143400) and *OsFerroportin* (TraesCS4D02G323100) genes on chromosomes 7 (OrMQTL_14) and 6 (OrMQTL_15) of rice which are related to Zn and Fe transport/homeostasis (Morrissey and Guerinot, 2009; Bashir et al., 2011; Alagarasan et al., 2017) (Table 5).

More intriguingly, the syntenic regions of the wheat MQTL_7B_3 on both chromosome 6 of rice (OrMQTL_10) and chromosome 5 of maize, harbored the rice (*OsMAPK4, Os06g0699400)* and maize (ZmMPK5, Zm00001d014658) and orthologous gene in wheat (TraesCS7B02G322900). The mitogen-activated protein kinase (MAPK) cascades play important roles in regulating plant growth (PH), development, and stress responses (Zhu et al., 2020). The maize *ZmMPK5* is induced by various stimuli, involved in defense signaling pathways in various abiotic/biotic stress, confers tolerance to stresses (Zhang A. et al., 2010; Zhang et al., 2014), and subsequently leads to enhancement of plant growth and yield. Due to the key role of *OsMAPK4* in plant growth, grain development (Liu et al., 2018; Chen et al., 2021), and subsequently in GY, the results of the current syntenic analysis suggest the same function for *TraesCS7B02G322900* and *Zm00001d014658* genes located in OrMQTL_10 interval (Figure 7; Table 5; Supplementary Table 7).

## Conclusion

The results of this study introduced several novel meta-quantitative trait loci (MQTLs) for improving wheat in multi-purpose breeding programs by identifying key genomic regions associated with agronomic performance, grain quality traits, and micronutrients content. The results of our MQTL analysis have significantly increased the power and precision of our ability to map wheat traits and will provide greater resolution for future fine mapping and marker development. Importantly, our data identify co-localization between grain yield (GY) QTLs with grain Zn content (GZnC), grain Fe content (GFeC), and grain protein content (GPC) QTLs that suggest an opportunity for simultaneous breeding for these traits. This study also provides an example for the value of comparative analysis between evolutionarily close cereal species for the identification of genomic regions and candidate genes (CGS) controlling quantitative traits. Our finding shows the utility of MQTL analysis for refining the location of genomic regions associated with a variety of traits and helps understand how their relative map positions can be exploited for crop improvement. Overall, these findings can lead to both increased selection efficiency and accuracy for breeding by providing the basis for marker development in a marker-assisted selection (MAS) program and for identifying a novel source of variation through allele mining efforts in genetic resource collections. Lastly, these refined MQTLs provide the basis for further focus on the genetic mechanisms controlling micronutrients, GY, and quality traits.

## Data Availability Statement

The original contributions presented in the study are included in the article/Supplementary Material, further inquiries can be directed to the corresponding author.

## Author Contributions

NS conducted bioinformatics analysis and wrote the draft of the article. BH conceived and designed the project, the bioinformatics analysis, and complemented the writing of the article. AT helped in bioinformatics analysis. CR helped with reviewing and providing critical advice on the article. All authors have read and approved the manuscript.

## Conflict of Interest

The authors declare that the research was conducted in the absence of any commercial or financial relationships that could be construed as a potential conflict of interest.

## Publisher's Note

All claims expressed in this article are solely those of the authors and do not necessarily represent those of their affiliated organizations, or those of the publisher, the editors and the reviewers. Any product that may be evaluated in this article, or claim that may be made by its manufacturer, is not guaranteed or endorsed by the publisher.

## Supplementary Material

Supplementary Figure 1 The location of detected MQTLs with 95% confidence interval associated with quantitative traits in wheat chromosomes. The Lines on the left side of the linkage groups indicate the confidence interval (CI) of QTLs. The colored boxes on each linkage groups represent MQTLs region and the colors inside the vertical lines on the left of illustrate the best model of MQTLs. The molecular markers and their genetic distance (cM) over linkage groups are shown on the right side.

Supplementary Table 1 The information of wheat population used for meta- quantitative trait loci (MQTL) analysis.

Supplementary Table 2 The initial and projected QTL data on the genetic consensus map and identified MQTLs for the studied traits.

Supplementary Table 3 The information of QTLs in the detected MQTLs.

Supplementary Table 4 The list of uncovered candidate genes (CGS) and all annotated genes anchoring at each MQTL interval.

Supplementary Table 5 The list of rice orthologous genes at each MQTL interval.

Supplementary Table 6 The information of the chi-square test for co-localization frequency analysis of the QTLs of the tested traits in the detected MQTLs regions.

Supplementary Table 7 The list of orthologous genes located at the syntenic regions of detected ortho-MQTLs between wheat, maize, and rice.

## Abbreviations

AIC: akaike information criterion
AICc: corrected AIC
AWE: the average weight of evidence
BIC: Bayesian information criterion
CI: confidence interval
GCs: candidate genes
GO: gene ontology
GWAS: genome wide association studies
LOD: the logarithm of odds
MQTL: meta- quantitative trait loci
MTP: metal tolerance proteins
OrMQTL: orthologous MQTL
QTL: quantitative trait loci
SNP: single-nucleotide polymorphism.

## References

[B1] Abdollahi-Sisi N. Mohammadi S. A. Razeghi J. (2018). Mapping QTLs for grain yield components in bread wheat under well-watered and rain-fed conditions. J. Bio. Env. Sci. 13, 306–314.

[B2] Acuña-Galindo M. A. Mason R. E. Subramanian N. K. Hays D. B. (2015). Meta-analysis of wheat QTL regions associated with adaptation to drought and heat stress. Crop. Sci. 55, 477–492. 10.2135/cropsci2013.11.0793

[B3] Ahn S. Anderson J. A. Sorrells M. E. Tanksley S. D. (1993). Homoeologous relationships of rice, wheat and maize chromosomes. Mol. Gen. Genet. 241, 483–490. 10.1007/BF00279889 7903411

[B4] Alagarasan G. Dubey M. Aswathy K. S. Chandel G. (2017). Genome wide identification of orthologous ZIP genes associated with zinc and Iron translocation in Setaria italica. Front. Plant. Sci. 8:775. 10.3389/fpls.2017.00775 28555148 PMC5430159

[B5] An-Ming D. I. Jun L. I. Fa C. U. Chun-Hua Z. H. Hang-Yun M. A. Hong-Gang W. A. (2011). Mapping QTLs for yield related traits using two associated RIL populations of wheat. Zuo Wu Xue Bao 37, 1511–1524. 10.3724/SP.J.1006.2011.01511

[B6] Arcade A. Labourdette A. Falque M. Mangin B. Chardon F. Charcosset A.. (2004). BioMercator: integrating genetic maps and QTL towards discovery of candidate genes. Bioinformatics 20, 2324–2326. 10.1093/bioinformatics/bth230 15059820

[B7] Avni R. Oren L. Shabtay G. Assili S. Pozniak C. Hale I.. (2018). Genome based meta-QTL analysis of grain weight in tetraploid wheat identifies rare alleles of GRF4 associated with larger grains. Genes 9:636. 10.3390/genes9120636 30562998 PMC6315823

[B8] Azadi A. Mardi M. Hervan E. M. Mohammadi S. A. Moradi F. Tabatabaee M. T.. (2014). QTL mapping of yield and yield components under normal and salt-stress conditions in bread wheat (*Triticum aestivum* L.). Plant Mol. Biol. Rep. 33, 102–120. 10.1007/s11105-014-0726-0

[B9] Badakhshan H. Moradi N. Mohammadzadeh H. Zakeri M. R. (2013). Genetic variability analysis of grains Fe, Zn and beta-carotene concentration of prevalent wheat varieties in Iran. Int. J. Agri. Crop Sci. 6:57.

[B10] Badji A. Otim M. Machida L. Odong T. Kwemoi D. B. Okii D.. (2018). Maize combined insect resistance genomic regions and their co-localization with Cell Wall constituents revealed by tissue-specific QTL meta-analyses. Front. Plant Sci. 9:895. 10.3389/fpls.2018.00895 30026746 PMC6041972

[B11] Bashir K. Ishimaru Y. Shimo H. Nagasaka S. Fujimoto M. Takanashi H.. (2011). The rice mitochondrial iron transporter is essential for plant growth. Nat. Commun. 2, 1–7. 10.1038/ncomms1326 21610725 PMC3113228

[B12] Belzile F. Abed A. Torkamaneh D. (2020). Time for a paradigm shift in the use of plant genetic resources. Genome 63, 189–194. 10.1139/gen-2019-0141 31825685

[B13] Bertoldi D. Tassoni A. Martinelli L. Bagni N. (2004). Polyamines and somatic embryogenesis in two Vitis vinifera cultivars. Physiol. Plant. 120, 657–666. 10.1111/j.0031-9317.2004.0282.x 15032828

[B14] Bethke P. C. Gubler F. Jacobsen J. V. Jones R. L. (2004). Dormancy of Arabidopsis seeds and barley grains can be broken by nitric oxide. Planta 219, 847–855. 10.1007/s00425-004-1282-x 15133666

[B15] Bezant H. Laurie D. A. Pratchett N. Chojecki J. Kearsey M. J. (1997). Mapping of QTL controlling NIR predicted hot water extract and grain nitrogen content in a spring barley cross using marker-regression. Plant Breed. 116, 141–145. 10.1111/j.1439-0523.1997.tb02168.x

[B16] Bhatta M. Baenziger P. Waters B. Poudel R. Belamkar V. Poland J.. (2018). Genome-wide association study reveals novel genomic regions associated with 10 grain minerals in synthetic hexaploid wheat. Int. J. Mol. Sci. 19:3237. 10.3390/ijms19103237 30347689 PMC6214031

[B17] Bhusal N. Sarial A. K. Sharma P. Sareen S. (2017). Mapping QTLs for grain yield components in wheat under heat stress. PLoS ONE 12:e0189594. 10.1371/journal.pone.0189594 29261718 PMC5736223

[B18] Black R. E. Victora C. G. Walker S. P. Bhutta Z. A. Christian P. de Onis M.. (2013). Maternal and child undernutrition and overweight in low-income and middle-income countries. Lancet 382, 427–451. 10.1016/S0140-6736(13)60937-X 23746772

[B19] Braun H. J. Atlin G. Payne T. (2010). “Multi-location testing as a tool to identify plant response to global climate change,” in Climate change and crop production, ed. M. P. Reynolds (CABI Press, Oxford), 115–138. 10.1079/9781845936334.0115

[B20] Chen B. X. Li W. Y. Gao Y. T. Chen Z. J. Zhang W. N. Liu Q. J.. (2016). Involvement of polyamine oxidase-produced hydrogen peroxide during coleorhiza-limited germination of rice seeds. Front. Plant. Sci. 7:1219. 10.3389/fpls.2016.01219 27570530 PMC4981591

[B21] Chen J. Wang L. Yuan M. (2021). Update on the roles of rice MAPK cascades. Int. J. Mol. Sci. 22:1679. 10.3390/ijms22041679 33562367 PMC7914530

[B22] Cheng S. Zhou D. X. Zhao Y. (2016). WUSCHEL-related homeobox gene WOX11 increases rice drought resistance by controlling root hair formation and root system development. Plant Signal Behav. 11:e1130198. 10.1080/15592324.2015.1130198 26689769 PMC4883865

[B23] Cona A. Moreno S. Cenci F. Federico R. Angelini R. (2005). Cellular re-distribution of flavin-containing polyamine oxidase in differentiating root and mesocotyl of *Zea mays* L. seedlings. Planta 221, 265–276. 10.1007/s00425-004-1435-y 15578214

[B24] Cooper M. Woodruff D. R. Eisemann R. L. Brennan P. S. DeLacy I. H. (1995). A selection strategy to accommodate genotype-by-environment interaction for grain yield of wheat: managed-environments for selection among genotypes. Theor. Appl. Genet. 90, 492–502. 10.1007/BF00221995 24173943

[B25] Crespo-Herrera L. A. Velu G. Singh R. P. (2016). Quantitative trait loci mapping reveals pleiotropic effect for grain iron and zinc concentrations in wheat. Ann. Appl. Biol. 169, 27–35. 10.1111/aab.12276

[B26] Danan S. Veyrieras J. B. Lefebvre V. (2011). Construction of a potato consensus map and QTL meta-analysis offer new insights into the genetic architecture of late blight resistance and plant maturity traits. BMC Plant Biol. 11:16. 10.1186/1471-2229-11-16 21247437 PMC3037844

[B27] De-la-Pena C. Galaz-Avalos R. M. Loyola-Vargas V. M. (2008). Possible role of light and polyamines in the onset of somatic embryogenesis of *Coffea canephora*. Mol. Biotechnol. 39, 215–224. 10.1007/s12033-008-9037-8 18228163

[B28] Delfino P. Zenoni S. Imanifard Z. Tornielli G. B. Bellin D. (2019). Selection of candidate genes controlling veraison time in grapevine through integration of meta-QTL and transcriptomic data. BMC Genomics 20, 1–19. 10.1186/s12864-019-6124-0 31615398 PMC6794750

[B29] Deng L. Li L. Zhang S. Shen J. Li S. Hu S.. (2017). Suppressor of rid1 (SID1) shares common targets with RID1 on florigen genes to initiate floral transition in rice. PLoS Genet. 13:e1006642. 10.1371/journal.pgen.1006642 28234896 PMC5345856

[B30] Deng X. Han X. Yu S. Liu Z. Guo D. He Y.. (2020). OsINV3 and its homolog, OsINV2, control grain size in rice. Int. J. Mol. Sci. 21:2199. 10.3390/ijms21062199 32209971 PMC7139340

[B31] Dixit S. Singh U. M. Abbai R. Ram T. Singh V. K. Paul A.. (2019). Identification of genomic region (s) responsible for high iron and zinc content in rice. Sci. Rep. 9:8136. 10.1038/s41598-019-43888-y 31148549 PMC6544658

[B32] El-Feki W. M. Byrne P. F. Reid S. D. Haley S. D. (2018). Mapping quantitative trait loci for agronomic traits in winter wheat under different soil moisture levels. Agronomy 8:133. 10.3390/agronomy8080133

[B33] Fabian D. Flatt T. (2012). Life history evolution. Nat. Edu. Knowl. 3:24.

[B34] Feng B. Chen K. Cui Y. Wu Z. Zheng T. Zhu Y.. (2018). Genetic dissection and simultaneous improvement of drought and low nitrogen tolerances by designed QTL pyramiding in rice. Front. Plant. Sci. 9:306. 10.3389/fpls.2018.00306 29593764 PMC5855007

[B35] Fernando N. Panozzo J. Tausz M. Norton R. Fitzgerald G. Seneweera S. (2012). Rising atmospheric CO2 concentration affects mineral nutrient and protein concentration of wheat grain. Food. Chem. 133, 1307–1311. 10.1016/j.foodchem.2012.01.105 25306370

[B36] Feuillet C. Keller B. (1999). High gene density is conserved at syntenic loci of small and large grass genomes. Proc. Natl. Acad. Sci. USA. 96, 8265–8270. 10.1073/pnas.96.14.8265 10393983 PMC22223

[B37] Flatt T. Heyland A. (2011). Mechanisms of Life History Evolution: the Genetics and Physiology of Life History Traits and Trade-Offs. Oxford: Oxford University Press. 10.1093/acprof:oso/9780199568765.001.0001

[B38] Flint J. Mackay T. F. (2009). Genetic architecture of quantitative traits in mice, flies, and humans. Genome Res. 19, 23–733. 10.1101/gr.086660.108 19411597 PMC3647534

[B39] Gahlaut V. Jaiswal V. Tyagi B. S. Singh G. Sareen S. Balyan H. S.. (2017). QTL mapping for nine drought-responsive agronomic traits in bread wheat under irrigated and rain-fed environments. PLoS ONE 12:e0182857. 10.1371/journal.pone.0182857 28793327 PMC5550002

[B40] Gale M. D. Devos K. M. (1998). Comparative genetics in the grasses. Proc. Natl. Acad. Sci. USA. 95, 1971–1974. 10.1073/pnas.95.5.1971 9482816 PMC33824

[B41] Gaut B. S. (2002). Evolutionary dynamics of grass genomes. New Phytol. 154, 15–28. 10.1046/j.1469-8137.2002.00352.x

[B42] Gautam T. Dhillon G. S. Saripalli G. Singh V. P. Prasad P. Kaur S.. (2020). Marker-assisted pyramiding of genes/QTL for grain quality and rust resistance in wheat (*Triticum aestivum* L.). Mol. Breed. 40, 1–14. 10.1007/s11032-020-01125-9

[B43] Giancaspro A. Giove S. L. Zacheo S. A. Blanco A. Gadaleta A. (2019). Genetic variation for protein content and yield-related traits in a durum population derived from an inter-specific cross between hexaploid and tetraploid wheat cultivars. Front. Plant Sci. 10:1509. 10.3389/fpls.2019.01509 31824537 PMC6883369

[B44] Goel S. Singh K. Singh B. Grewal S. Dwivedi N. Alqarawi A. A.. (2019). Analysis of genetic control and QTL mapping of essential wheat grain quality traits in a recombinant inbred population. PLoS ONE 14:e0200669. 10.1371/journal.pone.0200669 30840619 PMC6402682

[B45] Goffinet B. Gerber S. (2000). Quantitative trait loci: a meta-analysis. Genetics 155, 463–473. 10.1093/genetics/155.1.463 10790417 PMC1461053

[B46] Gokidi Y. Bhanu A. N. Chandra K. Singh M. N. Hemantaranjan A. (2017). Allele mining–an approach to discover allelic variation in crops. J. Plant Sci. Res. 33, 167–180.

[B47] Guo Y. Du Z. Chen J. Zhang Z. (2017). QTL mapping of wheat plant architectural characteristics and their genetic relationship with seven QTLs conferring resistance to sheath blight. PLoS ONE 12:e0174939. 10.1371/journal.pone.0174939 28384183 PMC5383044

[B48] Gustin J. L. Zanis M. J. Salt D. E. (2011). Structure and evolution of the plant cation diffusion facilitator family of ion transporters. BMC Evol. Biol. 11:76. 10.1186/1471-2148-11-76 21435223 PMC3073911

[B49] Hanocq E. Laperche A. Jaminon O. Lainé A. L. Le Gouis J. (2007). Most significant genome regions involved in the control of earliness traits in bread wheat, as revealed by QTL meta-analysis. Theor. Appl. Genet. 114, 569–584. 10.1007/s00122-006-0459-z 17171391

[B50] Hedden P. (2003). The genes of the Green Revolution. Trends Genet. 19, 5–9. 10.1016/S0168-9525(02)00009-4 12493241

[B51] Hong Y. Zhang H. Huang L. Li D. Song F. (2016). Overexpression of a stress-responsive NAC transcription factor gene ONAC022 improves drought and salt tolerance in rice. Front. Plant Sci. 7:4. 10.3389/fpls.2016.00004 26834774 PMC4722120

[B52] Hong Z. Ueguchi-Tanaka M. Fujioka S. Takatsuto S. Yoshida S. Hasegawa Y.. (2005). The rice brassinosteroid-deficient dwarf2 mutant, defective in the rice homolog of Arabidopsis DIMINUTO/DWARF1, is rescued by the endogenously accumulated alternative bioactive brassinosteroid, dolichosterone. Plant Cell 17, 2243–2254. 10.1105/tpc.105.030973 15994910 PMC1182486

[B53] Hu P. Zheng Q. Luo Q. Teng W. Li H. Li B.. (2020). Genome-wide association study of yield and related traits in common wheat under salt-stress conditions. BMC Plant Biol. 21:27. 10.1186/s12870-020-02799-1 33413113 PMC7792188

[B54] Itoh H. Ueguchi-Tanaka M. Sakamoto T. Kayano T. Tanaka H. Ashikari M.. (2002). Modification of rice plant height by suppressing the height-controlling gene, D18, in rice. Breed. Sci. 52, 215–218. 10.1270/jsbbs.52.215

[B55] Izawa T. Oikawa T. Sugiyama N. Tanisaka T. Yano M. Shimamoto K. (2002). Phytochrome mediates the external light signal to repress FT orthologs in photoperiodic flowering of rice. Genes Dev. 16, 2006–2020. 10.1101/gad.999202 12154129 PMC186415

[B56] Izquierdo P. Astudillo C. Blair M. W. Iqbal A. M. Raatz B. Cichy K. A. (2018). Meta-QTL analysis of seed iron and zinc concentration and content in common bean (*Phaseolus vulgaris* L.). Theor. Appl. Genet. 131, 1645–1658. 10.1007/s00122-018-3104-8 29752522

[B57] Jamil M. Ali A. Gul A. Ghafoor A. Napar A. A. Ibrahim A. M.. (2019). Genome-wide association studies of seven agronomic traits under two sowing conditions in bread wheat. BMC Plant Biol. 19:149. 10.1186/s12870-019-1754-6 31003597 PMC6475106

[B58] Kakkar R. K. Sawhney V. K. (2002). Polyamine research in plants—a changing perspective. Physiol. Plant. 116, 281–292. 10.1034/j.1399-3054.2002.1160302.x

[B59] Khahani B. Tavakol E. Shariati V. Fornara F. (2020). Genome wide screening and comparative genome analysis for meta-QTLs, ortho-MQTLs and candidate genes controlling yield and yield-related traits in rice. BMC Genomics 21, 1–24. 10.1186/s12864-020-6702-1 32272882 PMC7146888

[B60] Khush G. S. Lee S. Cho J. I. Jeon J. S. (2012). Biofortification of crops for reducing malnutrition. Plant Biotechnol. Rep. 6, 195–202. 10.1007/s11816-012-0216-5

[B61] Kolde R. (2013). pheatmap: Pretty Heatmaps. R package version 1.0.12. Available online at: http://CRAN.R-project.org/package=pheatmap (accessed December 26, 2018).

[B62] Kong W. Zhang Y. Deng X. Zhang C. Li Y. (2019). Comparative genomic and transcriptomic analyses suggests the evolutionary dynamic of gh3 genes in gramineae crops. Front. Plant Sci. 10:1297. 10.3389/fpls.2019.01297 31681387 PMC6803601

[B63] Krishnappa G. Singh A. M. Chaudhary S. Ahlawat A. K. Singh S. K. Shukla R. B.. (2017). Molecular mapping of the grain iron and zinc concentration, protein content and thousand kernel weight in wheat (*Triticum aestivum* L.). PLoS ONE 12:e0174972. 10.1371/journal.pone.0174972 28384292 PMC5383102

[B64] Kumar A. Saripalli G. Jan I. Kumar K. Sharma P. K. Balyan H. S.. (2020). Meta-QTL analysis and identification of candidate genes for drought tolerance in bread wheat (*Triticum aestivum* L.). Physiol. Mol. Biol. Plants 26, 1713–1725. 10.1007/s12298-020-00847-6 32801498 PMC7415061

[B65] Kumar G. R. Sakthivel K. Sundaram R. M. Neeraja C. N. Balachandran S. M. Rani N. S.. (2010). Allele mining in crops: prospects and potentials. Biotechnol. Adv. 28, 451–461. 10.1016/j.biotechadv.2010.02.007 20188810

[B66] Kumar S. Palve A. Joshi C. Srivastava R. K. (2019). Crop biofortification for iron (Fe), zinc (Zn) and vitamin A with transgenic approaches. Heliyon 5:e01914. 10.1016/j.heliyon.2019.e01914 31338452 PMC6579847

[B67] Kumari R. Kumar P. Sharma V. K. Kumar H. (2018). Allele mining for crop improvement. Int. J. Pure Appl. Biosci. 6, 1456–1465. 10.18782/2320-7051.6073

[B68] Lehner K. R. Taylor I. McCaskey E. N. Jain R. Ronald P. C. Goldman D. I.. (2018). A histidine kinase gene is required for large radius root tip circumnutation and surface exploration in rice. bioRxiv. 437012. 10.1101/437012

[B69] Lei L. Zheng H. L. Wang J. G. Liu H. L. Sun J. Zhao H. W.. (2018). Genetic dissection of rice (*Oryza sativa* L.) tiller, plant height, and grain yield based on QTL mapping and metaanalysis. Euphytica 214, 1–7. 10.1007/s10681-018-2187-2

[B70] Leung H. Raghavan C. Zhou B. Oliva R. Choi I. R. Lacorte V.. (2015). Allele mining and enhanced genetic recombination for rice breeding. Rice 8:34. 10.1186/s12284-015-0069-y 26606925 PMC4659784

[B71] Li F. Wen W. Liu J. Zhang Y. Cao S. He Z.. (2019). Genetic architecture of grain yield in bread wheat based on genome-wide association studies. BMC Plant Biol. 19:168. 10.1186/s12870-019-1781-3 31035920 PMC6489268

[B72] Li Q. Pan Z. Gao Y. Li T. Liang J. Zhang Z.. (2020). Quantitative trait locus (QTLs) mapping for quality traits of wheat based on high density genetic map combined with bulked segregant analysis RNA-seq (BSR-Seq) indicates that the basic 7S globulin gene is related to falling number. Front. Plant Sci. 11:1918. 10.3389/fpls.2020.600788 33424899 PMC7793810

[B73] Li W. T. Liu C. Liu Y. X. Pu Z. E. Dai S. F. Wang J. R.. (2013). Meta-analysis of QTL associated with tolerance to abiotic stresses in barley. Euphytica 189, 31–49. 10.1007/s10681-012-0683-3

[B74] Li X. Xu X. Liu W. Li X. Yang X. Ru Z.. (2020). Dissection of superior alleles for yield-related traits and their distribution in important cultivars of wheat by association mapping. Front. Plant Sci. 11:175. 10.3389/fpls.2020.00175 32194592 PMC7061769

[B75] Liang Y. Zhang K. Zhao L. Liu B. Meng Q. Tian J.. (2010). Identification of chromosome regions conferring dry matter accumulation and photosynthesis in wheat (*Triticum aestivum* L.). Euphytica 171, 145–156. 10.1007/s10681-009-0024-3

[B76] Lin H. Wang R. Qian Q. Yan M. Meng X. Fu Z.. (2009). DWARF27, an iron-containing protein required for the biosynthesis of strigolactones, regulates rice tiller bud outgrowth. Plant Cell 21, 1512–1525. 10.1105/tpc.109.065987 19470589 PMC2700539

[B77] Liszkay A. van der Zalm E. Schopfer P. (2004). Production of reactive oxygen intermediates (O_2_-, H_2_O_2_, and OH) by maize roots and their role in wall loosening and elongation growth. Plant Physiol. 136, 3114–3123. 10.1104/pp.104.044784 15466236 PMC523372

[B78] Liu J. Wu B. Singh R. P. Velu G. (2019). QTL mapping for micronutrients concentration and yield component traits in a hexaploid wheat mapping population. J. Cereal Sci. 88, 57–64. 10.1016/j.jcs.2019.05.008 33343062 PMC7729826

[B79] Liu L. Tong H. Xiao Y. Che R. Xu F. Hu B.. (2015). Activation of Big Grain1 significantly improves grain size by regulating auxin transport in rice. Proc. Natl. Acad. Sci. USA. 112, 11102–11107. 10.1073/pnas.1512748112 26283354 PMC4568269

[B80] Liu X. Feng Z. M. Zhou C. L. Ren Y. K. Mou C. L.. (2016). Brassinosteroid (BR) biosynthetic gene lhdd10 controls late heading and plant height in rice (*Oryza sativa* L.). Plant Cell Rep. 35, 357–368. 10.1007/s00299-015-1889-3 26518431

[B81] Liu X. Li J. Xu L. Wang Q. Lou Y. (2018). Expressing OsMPK4 impairs plant growth but enhances the resistance of rice to the striped stem borer Chilo suppressalis. Int. J. Mol. Sci. 19:1182. 10.3390/ijms19041182 29652796 PMC5979284

[B82] Löffler M. Schön C. C. Miedaner T. (2009). Revealing the genetic architecture of FHB resistance in hexaploid wheat (*Triticum aestivum* L.) by QTL meta-analysis. Mol. Breed. 23, 473–488. 10.1007/s11032-008-9250-y

[B83] Mahdi-Nezhad N. Kamali M. J. McIntyre C. L. Fakheri B. A. Omidi M. Masoudi B. (2019). Mapping QTLs with main and epistatic effect on Seri ‘M82× Babax ‘wheat population under salt stress. Euphytica 215:130. 10.1007/s10681-019-2450-1

[B84] Malik N. Dwivedi N. Singh A. K. Parida S. K. Agarwal P. Thakur J. K.. (2016). An integrated genomic strategy delineates candidate mediator genes regulating grain size and weight in rice. Sci. Rep. 6, 1–12. 10.1038/srep23253 27000976 PMC4802383

[B85] Martinez A. K. Soriano J. M. Tuberosa R. Koumproglou R. Jahrmann T. Salvi S. (2016). Yield QTLome distribution correlates with gene density in maize. Plant Sci. 242, 300–309. 10.1016/j.plantsci.2015.09.022 26566847

[B86] Marza F. Bai G. H. Carver B. F. Zhou W. C. (2006). Quantitative trait loci for yield and related traits in the wheat population Ning7840× Clark. Theor. Appl. Genet. 112, 688–698. 10.1007/s00122-005-0172-3 16369760

[B87] Mathew I. Shimelis H. Shayanowako A. I. T. Laing M. Chaplot V. (2019). Genome-wide association study of drought tolerance and biomass allocation in wheat. PLoS ONE 14:e0225383. 10.1371/journal.pone.0225383 31800595 PMC6892492

[B88] Michel S. Löschenberger F. Ametz C. Pachler B. Sparry E. Bürstmayr H. (2019). Combining grain yield, protein content and protein quality by multi-trait genomic selection in bread wheat. Theor. Appl. Genet. 132, 2767–2780. 10.1007/s00122-019-03386-1 31263910 PMC6763414

[B89] Minx P. Cordum H. Wilson R. (2005). Sequence, annotation, and analysis of synteny between rice chromosome 3 and diverged grass species. Genome Res. 15, 1284–1291. 10.1101/gr.3869505 16109971 PMC1199543

[B90] Mishra B. S. Jamsheer K. M. Singh D. Sharma M. Laxmi A. (2017). Genome-wide identification and expression, protein–protein interaction and evolutionary analysis of the seed plant-specific BIG GRAIN and BIG GRAIN LIKE gene family. Front. Plant Sci. 8:1812. 10.3389/fpls.2017.01812 29118774 PMC5660992

[B91] Mohan M. Nair S. Bhagwat A. Krishna T. G. Yano M. Bhatia C. R.. (1997). Genome mapping, molecular markers and marker-assisted selection in crop plants. Mol. Breed. 3, 87–103. 10.1023/A:100965191979224296899

[B92] Moin M. Bakshi A. Madhav M. S. Kirti P. B. (2017). Expression profiling of ribosomal protein gene family in dehydration stress responses and characterization of transgenic rice plants overexpressing RPL23A for water-use efficiency and tolerance to drought and salt stresses. Front. Chem. 5:97. 10.3389/fchem.2017.00097 29184886 PMC5694489

[B93] Monod C. Takahashi Y. Goldschmidt-Clermont M. Rochaix J. D. (1994). The chloroplast ycf8 open reading frame encodes a photosystem II polypeptide which maintains photosynthetic activity under adverse growth conditions. EMBO J. 13, 2747–2754. 10.1002/j.1460-2075.1994.tb06568.x 8026459 PMC395154

[B94] Moore G. Devos K. M. Wang Z. Gale M. D. (1995). Cereal genome evolution: grasses, line up and form a circle. Curr. Biol. 5, 737–739. 10.1016/S0960-9822(95)00148-5 7583118

[B95] Morey S. R. Hirose T. Hashida Y. Miyao A. Hirochika H. Ohsugi R.. (2018). Genetic evidence for the role of a rice vacuolar invertase as a molecular sink strength determinant. Rice 11:6. 10.1186/s12284-018-0201-x 29344835 PMC5772344

[B96] Morrissey J. Guerinot M. L. (2009). Iron uptake and transport in plants: the good, the bad, and the ionome. Chem. Rev. 109, 4553–4567. 10.1021/cr900112r 19754138 PMC2764373

[B97] Moschou P. N. Sanmartin M. Andriopoulou A. H. Rojo E. Sanchez-Serrano J. J. Roubelakis-Angelakis K. A. (2008). Bridging the gap between plant and mammalian polyamine catabolism: a novel peroxisomal polyamine oxidase responsible for a full back-conversion pathway in Arabidopsis. Plant Physiol. 147, 1845–1857. 10.1104/pp.108.123802 18583528 PMC2492618

[B98] Moschou P. N. Sarris P. F. Skandalis N. Andriopoulou A. H. Paschalidis K. A. Panopoulos N. J.. (2009). Engineered polyamine catabolism preinduces tolerance of tobacco to bacteria and oomycetes. Plant Physiol. 149, 1970–1981. 10.1104/pp.108.134932 19218362 PMC2663742

[B99] Murray C. J. Lopez A. D. (2013). Measuring the global burden of disease. N. Engl. J. Med. 369, 448–457. 10.1056/NEJMra1201534 23902484

[B100] Muthu V. Abbai R. Nallathambi J. Rahman H. Ramasamy S. Kambale R.. (2020). Pyramiding QTLs controlling tolerance against drought, salinity, and submergence in rice through marker assisted breeding. PLoS ONE 15:e0227421. 10.1371/journal.pone.0227421 31910435 PMC6946594

[B101] Nagelkerke N. J. (1991). A note on a general definition of the coefficient of determination. Biometrika 78, 691–692. 10.1093/biomet/78.3.691

[B102] Ogawa D. Yamamoto E. Ohtani T. Kanno N. Tsunematsu H. Nonoue Y.. (2018). Haplotype-based allele mining in the Japan-MAGIC rice population. Sci. Rep. 8:4379. 10.1038/s41598-018-22657-3 29531264 PMC5847589

[B103] Ogiso-Tanaka E. Matsubara K. Yamamoto S. I. Nonoue Y. Wu J. Fujisawa H.. (2013). Natural variation of the RICE FLOWERING LOCUS T 1 contributes to flowering time divergence in rice. PLoS ONE 8:e75959. 10.1371/journal.pone.0075959 24098411 PMC3788028

[B104] Ozawa S. Kobayashi T. Sugiyama R. Hoshida H. Shiina T. Toyoshima Y. (1997). Role of PSII-L protein (psbL gene product) on the electron transfer in photosystem II complex. 1. Over-production of wild-type and mutant versions of PSII-L protein and reconstitution into the PSII core complex. Plant Mol. Biol. 34, 151–161. 10.1023/A:1005800909495 9177321

[B105] Pachauri R. K. Allen M. R. Barros V. R. Broome J. Cramer W. Christ R.. (2014). Climate change 2014: synthesis report. Contribution of Working Groups I, II and III to the fifth assessment report of the Intergovernmental Panel on Climate Change (p. 151). IPCC.

[B106] Peleg Z. Cakmak I. Ozturk L. Yazici A. Jun Y. Budak H.. (2009). Quantitative trait loci conferring grain mineral nutrient concentrations in durum wheat × wild emmer wheat RIL population. Theor. Appl. Genet. 119, 353–369. 10.1007/s00122-009-1044-z 19407982

[B107] Pu C. X. Han Y. F. Zhu S. Song F. Y. Zhao Y. Wang C. Y.. (2017). The rice receptor-like kinases DWARF AND RUNTISH SPIKELET1 and 2 repress cell death and affect sugar utilization during reproductive development. Plant Cell 29, 70–89. 10.1105/tpc.16.00218 28082384 PMC5304344

[B108] Pu Z. E. Ma Y.u. He Q. Y. Chen G. Y. Wang J. R. Liu Y. X.. (2014). Quantitative trait loci associated with micronutrient concentrations in two recombinant inbred wheat lines. J. Integr. Agric. 13, 2322–2329. 10.1016/S2095-3119(13)60640-1

[B109] Quraishi U. M. Pont C. Ain Q. U. Flores R. Burlot L. Alaux M.. (2017). Combined genomic and genetic data integration of major agronomical traits in bread wheat (*Triticum aestivum* L.). Front. Plant Sci. 8:1843. 10.3389/fpls.2017.01843 29184557 PMC5694560

[B110] Ram H. Kaur A. Gandass N. Singh S. Deshmukh R. Sonah H.. (2019). Molecular characterization and expression dynamics of MTP genes under various spatio-temporal stages and metal stress conditions in rice. PLoS ONE 14:e0217360. 10.1371/journal.pone.0217360 31136613 PMC6538162

[B111] Ramya P. Chaubal A. Kulkarni K. Gupta L. Kadoo N. Dhaliwal H. S.. (2010). QTL mapping of 1000-kernel weight, kernel length, and kernel width in bread wheat (*Triticum aestivum* L.). J. Appl. Genet. 51, 421–429. 10.1007/BF03208872 21063060

[B112] Raza Q. Riaz A. Sabar M. Atif R. M. Bashir K. (2019). Meta-analysis of grain iron and zinc associated QTLs identified hotspot chromosomal regions and positional candidate genes for breeding biofortified rice. Plant Sci. 288:110214. 10.1016/j.plantsci.2019.110214 31521222

[B113] Rebetzke G. J. Condon A. G. Farquhar G. D. Appels R. Richards R. A. (2008). Quantitative trait loci for carbon isotope discrimination are repeatable across environments and wheat mapping populations. Theor. Appl. Genet. 118, 123–137. 10.1007/s00122-008-0882-4 18818897

[B114] Richmond T. A. Bleecker A. B. (1999). A defect in β-oxidation causes abnormal inflorescence development in Arabidopsis. Plant Cell 11, 1911–1923. 10.1105/tpc.11.10.1911 10521521 PMC144112

[B115] Rogalski M. Schöttler M. A. Thiele W. Schulze W. X. Bock R. (2008). Rpl33, a nonessential plastid-encoded ribosomal protein in tobacco, is required under cold stress conditions. Plant Cell 20, 2221–2237. 10.1105/tpc.108.060392 18757552 PMC2553612

[B116] Roshanzamir H. Kordenaeej A. Bostani A. (2013). Mapping QTLs related to Zn and Fe concentrations in bread wheat (*Triticum aestivum*) grain using microsatellite markers. Iranian J. Genet. Plant Breed. 2, 10–17.

[B117] Saini D. K. Devi P. Kaushik P. (2020). Advances in genomic interventions for wheat biofortification: a review. Agronomy 10:62. 10.3390/agronomy10010062

[B118] Salvi S. Tuberosa R. (2015). The crop QTLome comes of age. Curr. Opin. Biotechnol. 32, 179–185. 10.1016/j.copbio.2015.01.001 25614069

[B119] Semagn K. Beyene Y. Warburton M. L. Tarekegne A. Mugo S. Meisel B.. (2013). Meta-analyses of QTL for grain yield and anthesis silking interval in 18 maize populations evaluated under water-stressed and well-watered environments. BMC Genomics 14, 1–6. 10.1186/1471-2164-14-313 23663209 PMC3751468

[B120] Shahzad Z. Rouached H. Rakha A. (2014). Combating mineral malnutrition through iron and zinc biofortification of cereals. Compr. Rev. Food Sci. Food Saf. 13, 329–346. 10.1111/1541-4337.12063 33412655

[B121] Shariatipour N. Heidari B. (2020). “Genetic-based biofortification of staple food crops to meet zinc and iron deficiency-related challenges,” in Plant Micronutrients, Deficiency and Toxicity Management, ed. T. Aftab, K. R. Hakeem (Cham: Springer), 173–223. 10.1007/978-3-030-49856-6_8

[B122] Shukla S. Singh K. Patil R. V. Kadam S. Bharti S. Prasad P.. (2014). Genomic regions associated with grain yield under drought stress in wheat (*Triticum aestivum* L.). Euphytica 203, 449–467. 10.1007/s10681-014-1314-y

[B123] Simmonds N. W. (1995). The relation between yield and protein in cereal grain. J. Sci. Food Agric. 67, 309–315. 10.1002/jsfa.2740670306

[B124] Somers D. J. Isaac P. Edwards K. (2004). A high-density microsatellite consensus map for bread wheat (*Triticum aestivum* L.). Theor. Appl. Genet. 109, 1105–1114. 10.1007/s00122-004-1740-7 15490101

[B125] Soriano J. M. Alvaro F. (2019). Discovering consensus genomic regions in wheat for root-related traits by QTL meta-analysis. Sci. Rep. 9:10537. 10.1038/s41598-019-47038-2 31332216 PMC6646344

[B126] Sosnowski O. Charcosset A. Joets J. (2012). BioMercator V3: an upgrade of genetic map compilation and quantitative trait loci meta-analysis algorithms. Bioinformatics 28, 2082–2083. 10.1093/bioinformatics/bts313 22661647 PMC3400960

[B127] Spindel J. E. Begum H. Akdemir D. Collard B. Redoña E. Jannink J. L.. (2016). Genome-wide prediction models that incorporate *de novo* GWAS are a powerful new tool for tropical rice improvement. Heredity 116, 395–408. 10.1038/hdy.2015.113 26860200 PMC4806696

[B128] Stein A. J. (2010). Global impacts of human mineral malnutrition. Plant Soil 335, 133–154. 10.1007/s11104-009-0228-2

[B129] Swamy B. P. M. Kaladhar K. Anuradha K. Batchu A. K. Longvah T. Sarla N. (2018). QTL analysis for grain iron and zinc concentrations in two *O. nivara* derived backcross populations. Rice Sci. 25, 197–207. 10.1016/j.rsci.2018.06.003

[B130] Swamy B. P. M. Sarla N. (2011). Meta-analysis of yield QTLs derived from interspecific crosses of rice reveals consensus regions and candidate genes. Plant Mol. Biol. Rep. 29, 663–680. 10.1007/s11105-010-0274-1

[B131] Tamiru M. Undan J. R. Takagi H. Abe A. Yoshida K. Undan J. Q.. (2015). A cytochrome P450, OsDSS1, is involved in growth and drought stress responses in rice (*Oryza sativa* L.). Plant Mol. Biol. 88, 85–99. 10.1007/s11103-015-0310-5 25800365

[B132] Tiwari C. Wallwork H. Arun B. Mishra V. K. Velu G. Stangoulis J.. (2016). Molecular mapping of quantitative trait loci for zinc, iron and protein content in the grains of hexaploid wheat. Euphytica 207, 563–570. 10.1007/s10681-015-1544-7

[B133] Tiwari V. K. Rawat N. Chhuneja P. Neelam K. Aggarwal R. Randhawa G. S.. (2009). Mapping of quantitative trait loci for grain iron and zinc concentration in diploid A genome wheat. J. Hered. 100, 771–776. 10.1093/jhered/esp030 19520762

[B134] Truntzler M. Barriere Y. Sawkins M. C. Lespinasse D. Betran J. Charcosset A.. (2010). Meta-analysis of QTL involved in silage quality of maize and comparison with the position of candidate genes. Theor. Appl. Genet. 121, 1465–1482. 10.1007/s00122-010-1402-x 20658277

[B135] Velu G. Guzman C. Mondal S. Autrique J. E. Huerta J. Singh R. P. (2016). Effect of drought and elevated temperature on grain zinc and iron concentrations in CIMMYT spring wheat. J. Cereal Sci. 69, 182–186. 10.1016/j.jcs.2016.03.006

[B136] Velu G. Singh R. P. Crespo-Herrera L. Juliana P. Dreisigacker S. Valluru R.. (2018). Genetic dissection of grain zinc concentration in spring wheat for mainstreaming biofortification in CIMMYT wheat breeding. Sci. Rep. 8:13526. 10.1038/s41598-018-31951-z 30201978 PMC6131222

[B137] Velu G. Tutus Y. Gomez-Becerra H. F. Hao Y. Demir L. Kara R.. (2017). QTL mapping for grain zinc and iron concentrations and zinc efficiency in a tetraploid and hexaploid wheat mapping populations. Plant Soil 411, 81–99. 10.1007/s11104-016-3025-8

[B138] Veyrieras J. B. Goffinet B. Charcosset A. (2007). MetaQTL: a package of new computational methods for the meta-analysis of QTL mapping experiments. BMC Bioinform. 8:49. 10.1186/1471-2105-8-49 17288608 PMC1808479

[B139] Vij S. Giri J. Dansana P. K. Kapoor S. Tyagi A. K. (2008). The receptor-like cytoplasmic kinase (*OsRLCK*) gene family in rice: organization, phylogenetic relationship, and expression during development and stress. Mol. Plant 1, 732–750. 10.1093/mp/ssn047 19825577

[B140] Vijayalakshmi K. Fritz A. K. Paulsen G. M. Bai G. Pandravada S. Gill B. S. (2010). Modeling and mapping QTL for senescence-related traits in winter wheat under high temperature. Mol. Breed. 26, 163–175. 10.1007/s11032-009-9366-8

[B141] Wan Y. King R. Mitchell R. A. Hassani-Pak K. Hawkesford M. J. (2017). Spatiotemporal expression patterns of wheat amino acid transporters reveal their putative roles in nitrogen transport and responses to abiotic stress. Sci. Rep. 7:5461. 10.1038/s41598-017-04473-3 28710348 PMC5511167

[B142] Wang L. I. Cui F. A. Wang J. Jun L. I. Ding A. Zhao C.. (2012). Conditional QTL mapping of protein content in wheat with respect to grain yield and its components. J. Genet. 91, 303–312. 10.1007/s12041-012-0190-2 23271016

[B143] Wang P. Xing Y. Li Z. Yu S. (2012). Improving rice yield and quality by QTL pyramiding. Mol. Breed. 29, 903–913. 10.1007/s11032-011-9679-2

[B144] Wang Y. Huang Z. Deng D. Ding H. Zhang R. Wang S.. (2013). Meta-analysis combined with syntenic metaQTL mining dissects candidate loci for maize yield. Mol. Breed. 31, 601–614. 10.1007/s11032-012-9818-4

[B145] Wang Y. Shi C. Yang T. Zhao L. Chen J. Zhang N.. (2018). High-throughput sequencing revealed that microRNAs were involved in the development of superior and inferior grains in bread wheat. Sci. Rep. 8, 1–18. 10.1038/s41598-018-31870-z 30218081 PMC6138641

[B146] Wang Y. Xu J. Deng D. Ding H. Bian Y. Yin Z.. (2016). A comprehensive meta-analysis of plant morphology, yield, stay-green, and virus disease resistance QTL in maize (*Zea mays* L.). Planta 243, 459–471. 10.1007/s00425-015-2419-9 26474992

[B147] Wu C. You C. Li C. Long T. Chen G. Byrne M. E.. (2008). RID1, encoding a Cys2/His2-type zinc finger transcription factor, acts as a master switch from vegetative to floral development in rice. Proc. Natl. Acad. Sci. USA. 105, 12915–12920. 10.1073/pnas.0806019105 18725639 PMC2529042

[B148] Wu X. L. Hu Z. L. (2012). Meta-analysis of QTL mapping experiments. Methods Mol. Biol. 871, 145–171. 10.1007/978-1-61779-785-9_8 22565836

[B149] Wu Y. Zhao S. Li X. Zhang B. Jiang L. Tang Y.. (2018). Deletions linked to PROG1 gene participate in plant architecture domestication in Asian and African rice. Nat. Commun. 9, 1–10. 10.1038/s41467-018-06509-2 30297755 PMC6175861

[B150] Xiang K. Reid L. M. Zhang Z. M. Zhu X. Y. Pan G. T. (2012). Characterization of correlation between grain moisture and ear rot resistance in maize by QTL meta-analysis. Euphytica 183, 185–195. 10.1007/s10681-011-0440-z

[B151] Xu L. Zhao H. Ruan W. Deng M. Wang F. Peng J.. (2017). ABNORMAL INFLORESCENCE MERISTEM1 functions in salicylic acid biosynthesis to maintain proper reactive oxygen species levels for root meristem activity in rice. Plant Cell 29, 560–574. 10.1105/tpc.16.00665 28298519 PMC5385951

[B152] Xu Y. An D. Liu D. Zhang A. Xu H. Li B. (2012). Molecular mapping of QTLs for grain zinc, iron and protein concentration of wheat across two environments. Field Crops Res. 138, 57–62. 10.1016/j.fcr.2012.09.017

[B153] Xue W. Xing Y. Weng X. Zhao Y. Tang W. Wang L.. (2008). Natural variation in Ghd7 is an important regulator of heading date and yield potential in rice. Nat. Genet. 40, 761–767. 10.1038/ng.143 18454147

[B154] Yamburenko M. V. Kieber J. J. Schaller G. E. (2017). Dynamic patterns of expression for genes regulating cytokinin metabolism and signaling during rice inflorescence development. PLoS ONE 12:e0176060. 10.1371/journal.pone.0176060 28419168 PMC5395194

[B155] Yao R. Wang L. Li Y. Chen L. Li S. Du X.. (2018). Rice DWARF14 acts as an unconventional hormone receptor for strigolactone. J. Exp. Bot. 69, 2355–2365. 10.1093/jxb/ery014 29365172 PMC5913607

[B156] Yu Y. Ouyang Y. Yao W. (2017). shinyCircos: an R/Shiny application for interactive creation of Circos plot. Bioinformatics 34, 1229–1231. 10.1093/bioinformatics/btx763 29186362

[B157] Zhang A. Zhang J. Ye N. Cao J. Tan M. Zhang J.. (2010). ZmMPK5 is required for the NADPH oxidase-mediated self-propagation of apoplastic H_2_O_2_ in brassinosteroid-induced antioxidant defence in leaves of maize. J. Exp. Bot. 61, 4399–4411. 10.1093/jxb/erq243 20693409 PMC2955750

[B158] Zhang D. Jiang S. Pan J. Kong X. Zhou Y. Liu Y.. (2014). The overexpression of a maize mitogen-activated protein kinase gene (ZmMPK5) confers salt stress tolerance and induces defence responses in tobacco. Plant Biol. 16, 558–570. 10.1111/plb.12084 23952812

[B159] Zhang H. Zhang J. Yan J. Gou F. Mao Y. Tang G.. (2017). Short tandem target mimic rice lines uncover functions of miRNAs in regulating important agronomic traits. Proc. Natl. Acad. Sci. USA. 114, 5277–5282. 10.1073/pnas.1703752114 28461499 PMC5441788

[B160] Zhang L. Y. Liu D. C. Guo X. L. Yang W. L. Sun J. Z. Wang D. W.. (2010). Genomic distribution of quantitative trait loci for yield and yield-related traits in common wheat. J. Integr. Plant. Biol. 52, 996–1007. 10.1111/j.1744-7909.2010.00967.x 20977657

[B161] Zhang M. Cui Y. Liu Y. H. Xu W. Sze S. H. Murray S. C.. (2020). Accurate prediction of maize grain yield using its contributing genes for gene-based breeding. Genomics 112, 225–236. 10.1016/j.ygeno.2019.02.001 30826444

[B162] Zhang M. Liu B. (2017). Identification of a rice metal tolerance protein OsMTP11 as a manganese transporter. PLoS ONE 12:e0174987. 10.1371/journal.pone.0174987 28394944 PMC5386239

[B163] Zhang T. Feng P. Li Y. Yu P. Yu G. Sang X.. (2018). VIRESCENT-ALBINO LEAF 1 regulates leaf colour development and cell division in rice. J. Exp. Bot. 69, 4791–4804. 10.1093/jxb/ery250 30102358 PMC6137968

[B164] Zhao H. Duan K. X. Ma B. Yin C. C. Hu Y. Tao J. J.. (2020). Histidine kinase MHZ1/OsHK1 interacts with ethylene receptors to regulate root growth in rice. Nat. Commun. 11, 1–13. 10.1038/s41467-020-14313-0 31980616 PMC6981129

[B165] Zhao X. Peng Y. Zhang J. Fang P. Wu B. (2018). Identification of QTLs and meta-QTLs for seven agronomic traits in multiple maize populations under well-watered and water-stressed conditions. Crop Sci. 58, 507–520. 10.2135/cropsci2016.12.0991

[B166] Zhao Y. Cheng S. Song Y. Huang Y. Zhou S. Liu X.. (2015). The interaction between rice *ERF3* and *WOX11* promotes crown root development by regulating gene expression involved in cytokinin signaling. Plant Cell 27, 2469–2483. 10.1105/tpc.15.00227 26307379 PMC4815106

[B167] Zhou W. Wang Y. Wu Z. Luo L. Liu P. Yan L.. (2016). Homologs of SCAR/WAVE complex components are required for epidermal cell morphogenesis in rice. J. Exp. Bot. 67, 4311–4323. 10.1093/jxb/erw214 27252469 PMC5301933

[B168] Zhu D. Chang Y. Pei T. Zhang X. Liu L. Li Y.. (2020). MAPK-like protein 1 positively regulates maize seedling drought sensitivity by suppressing ABA biosynthesis. Plant J. 102, 747–760. 10.1111/tpj.14660 31863495

[B169] Zilic S. Barac M. Pešic M. Dodig D. Ignjatovic-Micic D. (2011). Characterization of proteins from grain of different bread and durum wheat genotypes. Int. J. Mol. Sci. 12, 5878–5894. 10.3390/ijms12095878 22016634 PMC3189758

[B170] Zou Y. Liu X. Wang Q. Chen Y. Liu C. Qiu Y.. (2014). *OsRPK1*, a novel leucine-rich repeat receptor-like kinase, negatively regulates polar auxin transport and root development in rice. Biochim. Biophys. Acta 1840, 1676–1685. 10.1016/j.bbagen.2014.01.003 24412327

