# Supplementary material for: Comparative Genomic Analysis of Quantitative Trait Loci Associated With Micronutrient Contents, Grain Quality, and Agronomic Traits in Wheat (Triticum aestivum L.)
Source: Front Plant Sci. 2021 Oct 12;12:709817. doi: 10.3389/fpls.2021.709817 (PMC8546302; doi:10.3389/fpls.2021.709817)
Supplement: Supplementary Figure 1 — The location of detected MQTLs with 95% confidence interval associated with quantitative traits in wheat chromosomes. The Lines on the left side of the linkage groups indicate the confidence interval (CI) of QTLs. The colored boxes on each linkage groups represent MQTLs region and the colors inside the vertical lines on the left of illustrate the best model of MQTLs. The molecular markers and their genetic distance (cM) over linkage groups are shown on the right side. [file Data_Sheet_1.docx]

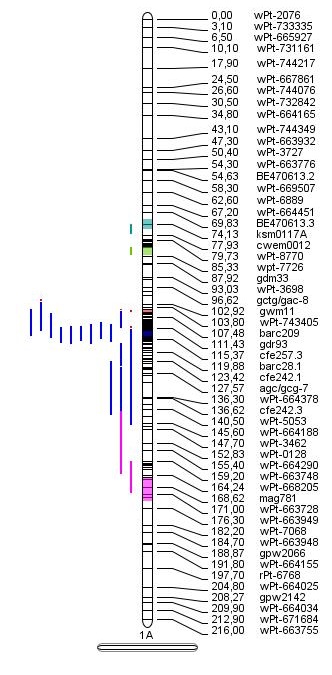

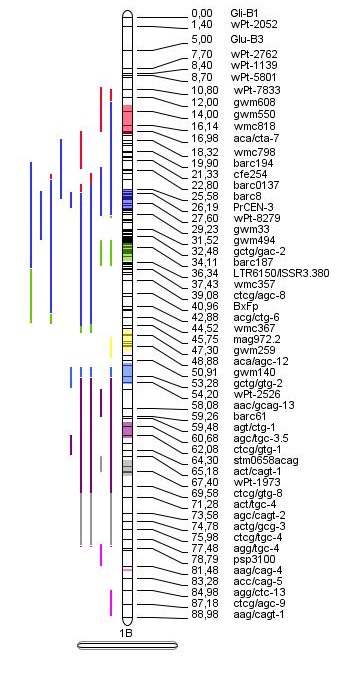

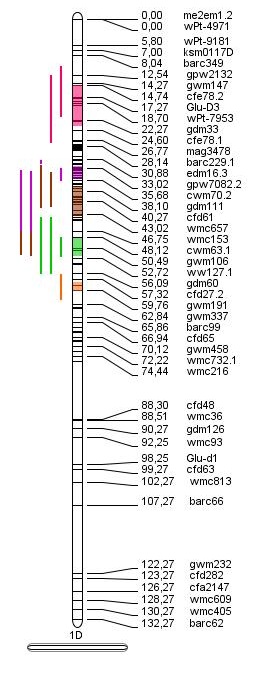


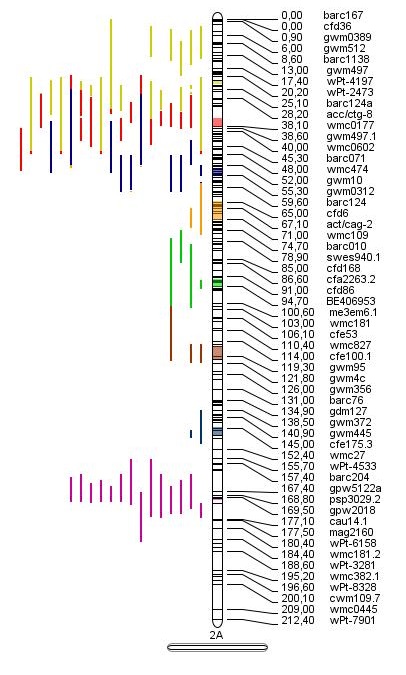

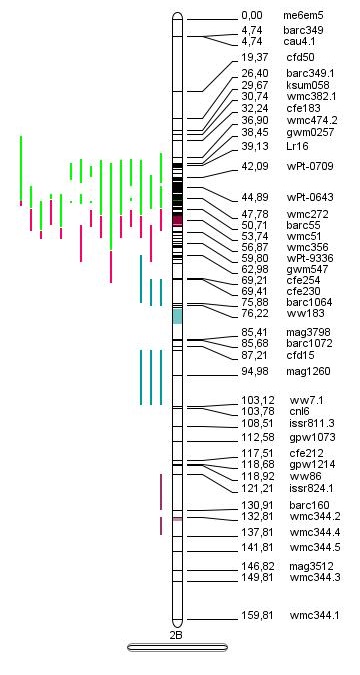

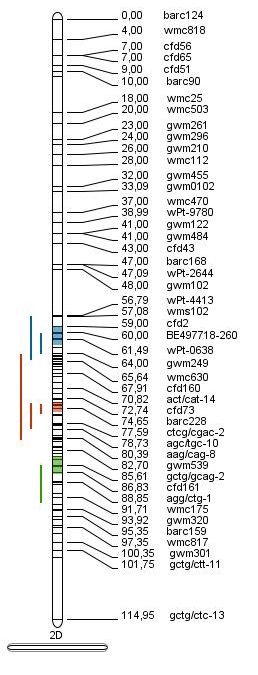


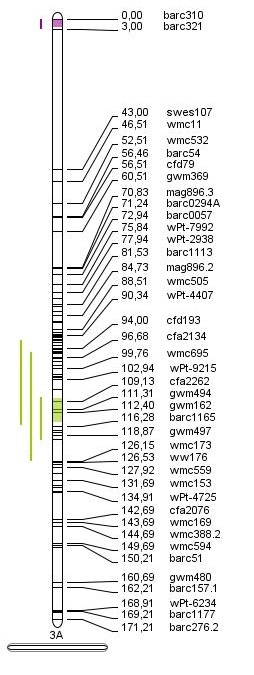

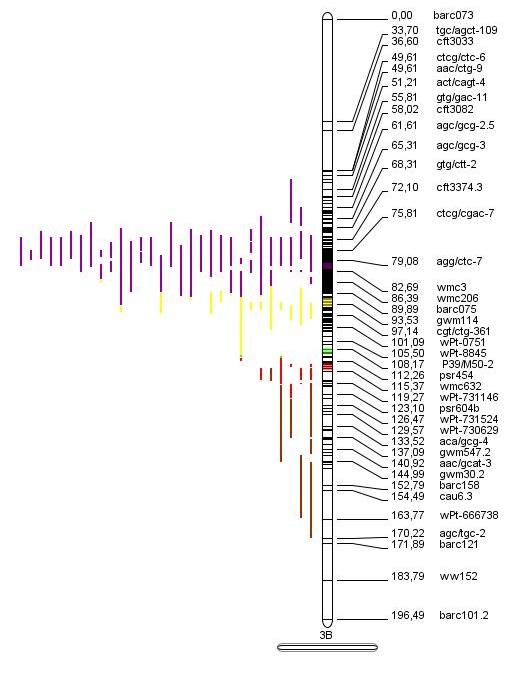

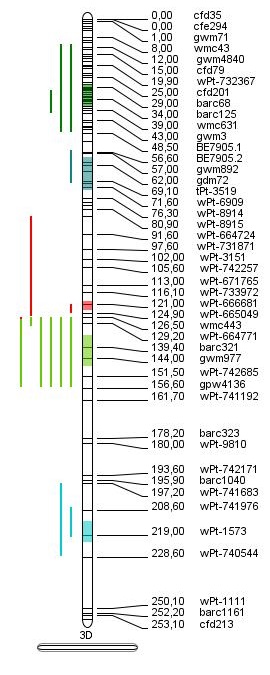


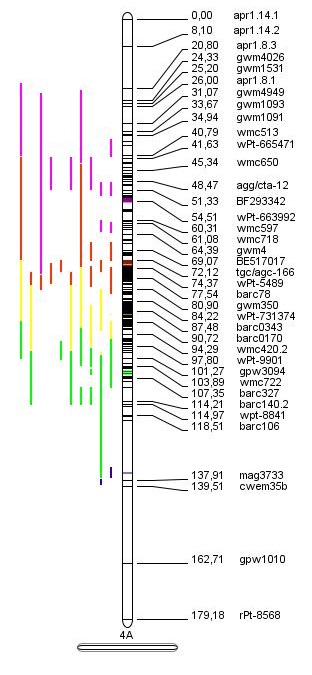

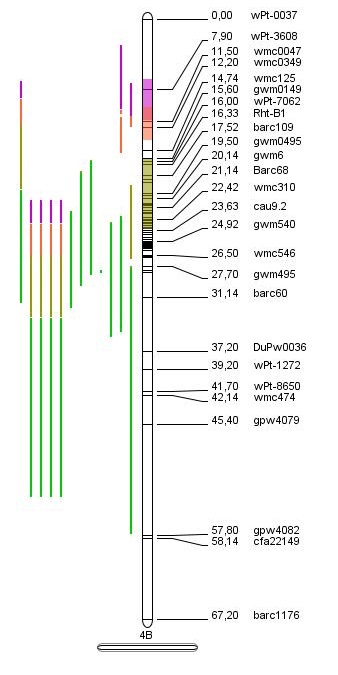

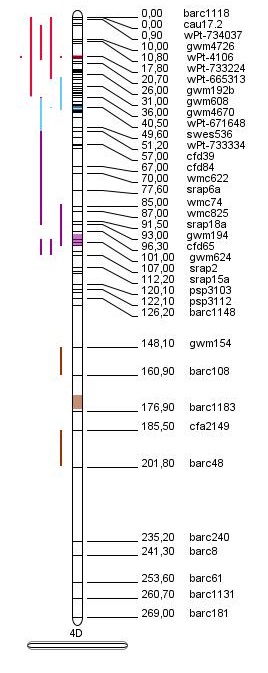


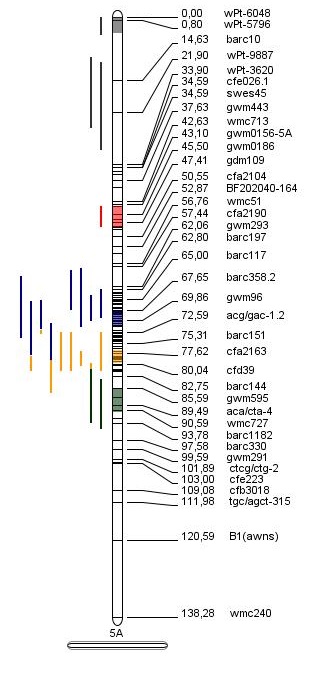

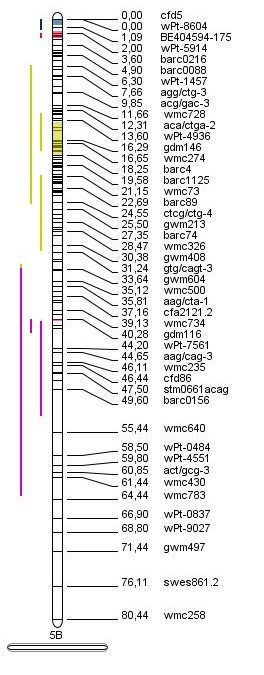

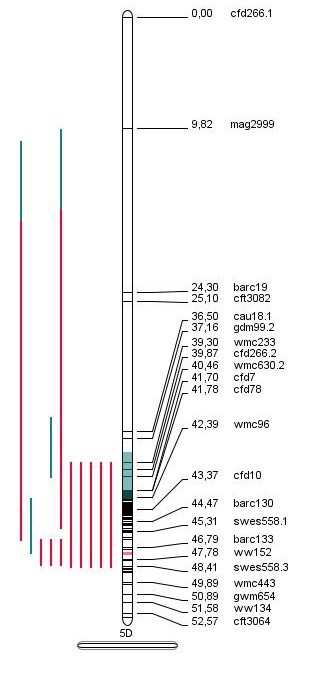


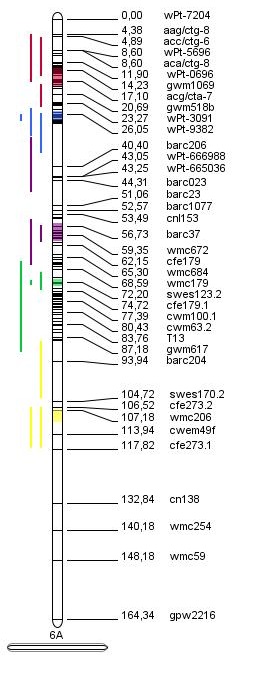

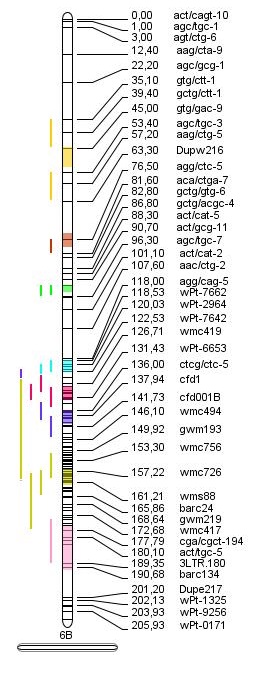

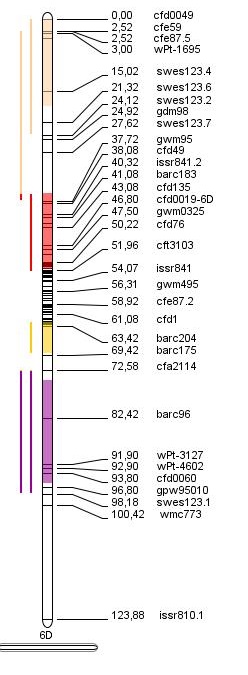


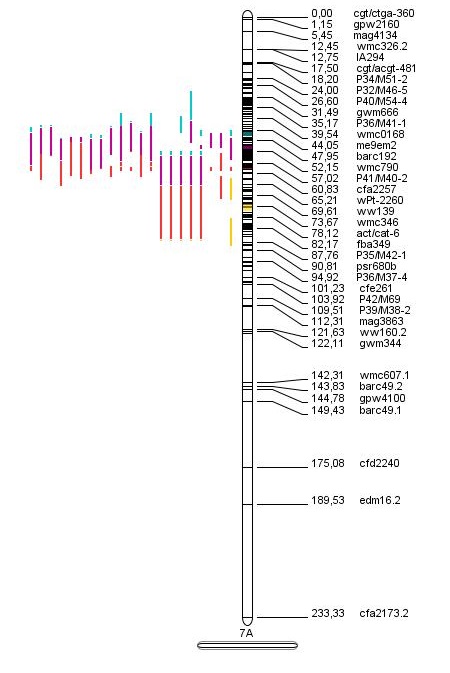

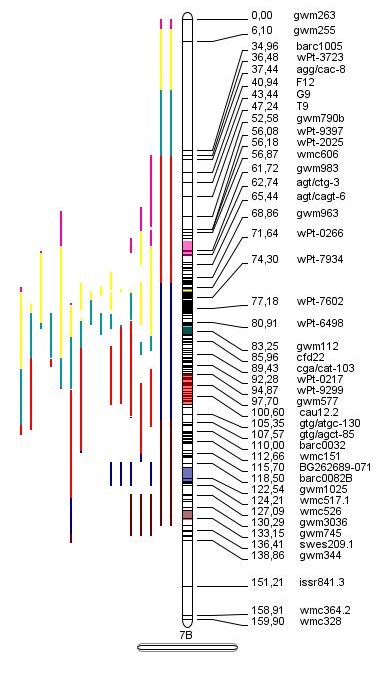

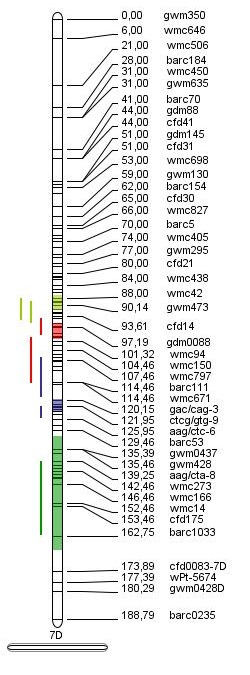


**Supplementary Figure 1.** The location of detected MQTLs with 95% confidence interval associated with quantitative traits in wheat chromosomes. The Lines on the left side of the linkage groups indicate the confidence interval (CI) of QTLs. The colored boxes on each linkage groups represent MQTLs region and the colors inside the vertical lines on the left of illustrate the best model of MQTLs. The molecular markers and their genetic distance (cM) over linkage groups are shown on the right side.
