# Supplementary material for: Comparative Genomic Analysis of Quantitative Trait Loci Associated With Micronutrient Contents, Grain Quality, and Agronomic Traits in Wheat (Triticum aestivum L.)
Source: Front Plant Sci. 2021 Oct 12;12:709817. doi: 10.3389/fpls.2021.709817 (PMC8546302; doi:10.3389/fpls.2021.709817)
Supplement: Supplementary Table 1 — The information of wheat population used for meta- quantitative trait loci (MQTL) analysis. [file Table_1.docx]

| **Supplementary Table 1.** Information of wheat population used for meta-QTL analysis. | | | | | | |
| --- | --- | --- | --- | --- | --- | --- |
| Population | Population Parents | Population Type | Population Size | Type of Marker | Trait^*^ | References |
| Pop_1 | Superhead#2 × Roshan | RIL | 186 | SSR, DArT | GY, NG, TKW, SL, SNS, SW, BY | Azadi et al., 2014 |
| Pop_2 | HD2808 × HUW510 | RIL | 397 | SSR | GWs, NG, TKW, GFD, GFR, GY | Bhusal et al., 2017 |
| Pop_3 | Weimai 8 × Yannong 19 | RIL | 229 | G-SSR, EST-SSR, ISSR, STS, SRAP, RAPD | SNS, NG, SN, TKW, GY | An-Ming et al., 2011 |
| Pop_4 | Weimai 8 ×Jimai 20 | RIL | 485 | G-SSR, EST-SSR, ISSR, STS, SRAP, RAPD | SNS, NG, SN, TKW, GY |  |
| Pop_5 | CO940610 × ‘Platte’ (PI 596297) | DH | 185 | SSR, STS, DArT | PH, LL, LW, SL, DH, DPM, GFD, GFR, AGB, HI, 200KW, GY | El-Feki et al., 2018 |
| Pop_6 | Excalibur × Kukri | DH | 92 | SSR, DArT | DA, DPM, GFD, PH, PT, GWe, TKW, GY | Gahlaut et al., 2017 |
| Pop_7 | WL711 (S308/Chris/Kalyansona) × C306 (RGB/CSL3//2/C591/3/C217/N14//C281) | RIL | 206 | SSR, EST-STS | GPC, SDS, HW, TKW, SD, WGC, DGC, FWA, DDT, DST, MTI, BDT, KH | Goel et al., 2019 |
| Pop_8 | Luke × AQ24788-83 | RIL | 266 | SSR, EST-SSR | DH, PH, FLH, IH, LH | Guo et al., 2017 |
| Pop_9 | WH542 × *Triticum dicoccon* PI94624*/Aegilops sqarrosa* [409]*//BCN* | RIL | 286 | SSR | GFe, GZn, GPC, TKW | Krishnappa et al., 2017 |
| Pop_10 | NW1014 × HUW468 | RIL | 106 | SSR | GL, GW, GL/GW, GPL, GAS, FFD, TKW | Kumari et al., 2018 |
| Pop_11 | Huapei 3 (HP3) × Yumai 57 (YM57) | DH | 168 | SSR, EST, ISSR | CDMA, LDMA, PDMA | Liang et al., 2010 |
| Pop_12 | Seri M82 × Babax | RIL | 169 | SSR, AFLP, DArT | DH, DA, BY, TKW, GY | Mahdi-Nezhad et al., 2019 |
| Pop_13 | Ning7840 × Clark | RIL | 132 | SSR, AFLP | GY, SN, KN, SW, DH, MDR, PH, SHS, LS, LY, SL | Marza et al., 2006 |
| Pop_14 | SHW-L1 × Chuanmai 32 | RIL | 171 | SSR, DArT | GSe, GFe, GZn, GCu, GMn | Pu et al., 2014 |
| Pop_15 | Chuanmai 42 × Chuannong 16 | RIL | 127 | SSR, SRAP | GSe, GFe, GZn, GMn |  |
| Pop_16 | Rye Selection 111 (RS) × Chinese Spring (CS) | RIL | 185 | SSR | TKW, KL, KW | Ramya et al., 2010 |
| Pop_17 | Cranbrook × Halberd | DH | 190 | SSR, DArT, Biochemical and Morphological Markers | CID | Rebetzke et al., 2008 |
| Pop_18 | Sunco × Tasman | DH | 161 | SSR, DArT, Biochemical and Morphological Markers | CID |  |
| Pop_19 | CD87 × Katepwa | DH | 190 | SSR, DArT, Biochemical and Morphological Markers | CID |  |
| Pop_20 | Tabassi × Taifun | RIL | 118 | SSR, Morphological Markers | GFe, GZn | Roshanzamir et al., 2013 |
| Pop_21 | WL711 ((S308/Chris)/Kalyansona) × C306 (RGB/CSL3// 2/C591/3/C217/N14//C281) | RIL | 206 | SSR, EST-STS | GY, BM, GN, TKW, TN, SN, HI, PH, DTF | Shukla et al., 2014 |
| Pop_22 | Iran #49 × Yecora Rojo | RIL | 149 | SSR, Retrotransposon Markers | GY, NG, TKW, PH, SL | Abdollahi-Sisi et al., 2018 |
| Pop_23 | *T. boeoticum* accession pau5088 × *T. monococcum* accession pau14087 | RIL | 93 | SSR, RFLP, Morphological Markers | GFe, GZn | Tiwari et al., 2009 |
| Pop_24 | Berkut × Krichauff | DH | 138 | SSR, DArT, *Vrn* gene linked marker | GZn, GFe, GPC | Tiwari et al., 2016 |
| Pop_25 | Adana99 × *Triticum sphaerococum* | RIL | 127 | DArT | GZn, ZnE, SHZn, GFe | Velu et al., 2017 |
| Pop_26 | Ventnor × Karl 92 | RIL | 101 | SSR, AFLP, EST | 75%G, 50%G, 25%G, MRS, TMRS, PGMS | Vijayalakshmi et al., 2010 |
| Pop_27 | Weimai 8 × Luohan 2 | RIL | 302 | G-SSR, EST-SSR, ISSR, STS, SRAP | GPC | Wang et al., 2012 |
|  | | | | | | |
| RIL, recombinant inbred line; DH, double haploid.  * The full name of assessed traits are displayed in Table 1. | | | | | | |
