# Supplementary material for: Comparative Genomic Analysis of Quantitative Trait Loci Associated With Micronutrient Contents, Grain Quality, and Agronomic Traits in Wheat (Triticum aestivum L.)
Source: Front Plant Sci. 2021 Oct 12;12:709817. doi: 10.3389/fpls.2021.709817 (PMC8546302; doi:10.3389/fpls.2021.709817)
Supplement: Supplementary Table 2 — The initial and projected QTL data on the genetic consensus map and identified MQTLs for the studied traits. [file Table_2.docx]

| **Supplementary Table 2.** Summary of trait data and number of QTLs and identified M-QTLs in each class of studied traits. The Agronomic traits represent traits that are mostly involved in the appearance form of the plant whilst the physiological traits refer to those traits that are involved in reactions inside the plant. | | | | | | | | |
| --- | --- | --- | --- | --- | --- | --- | --- | --- |
| Class of Traits * | Type of Traits † | Number of Individual QTL ‡ | Number of projected QTL in MQTL region |  | Class of Traits * | Type of Traits † | Number of Individual QTL ‡ | Number of projected QTL in MQTL region |
| 200KW | A | 5 | 0 |  | GY | A | 67 | 40 |
| 25%G | P | 4 | 2 |  | GZnC | M | 23 | 11 |
| 50%G | P | 4 | 4 |  | HI | A | 6 | 4 |
| 75%G | P | 4 | 4 |  | HW | A | 4 | 4 |
| AGB | A | 2 | 0 |  | KH | Q | 3 | 3 |
| BDT | Q | 2 | 1 |  | KL | A | 8 | 2 |
| BM | A | 4 | 4 |  | KW | A | 15 | 5 |
| BY | A | 4 | 2 |  | LDMA | P | 7 | 5 |
| CDMA | P | 3 | 1 |  | LL | A | 4 | 0 |
| CID | P | 12 | 6 |  | LS | A | 3 | 2 |
| DA | A | 12 | 10 |  | LW | A | 1 | 0 |
| DDT | Q | 2 | 2 |  | LY | P | 6 | 4 |
| DGC | Q | 1 | 1 |  | MDR | A | 3 | 2 |
| DH | A | 12 | 6 |  | MRS | P | 5 | 4 |
| DPM | A | 13 | 10 |  | MTI | Q | 1 | 1 |
| DST | Q | 2 | 2 |  | NG | A | 60 | 40 |
| DTF | A | 2 | 1 |  | PDMA | P | 5 | 3 |
| FFD | A | 2 | 0 |  | PGMS | P | 4 | 3 |
| FLH | A | 1 | 0 |  | PH | A | 28 | 17 |
| FWA | Q | 3 | 2 |  | PLH | A | 1 | 0 |
| GAS | A | 3 | 0 |  | PT | A | 5 | 5 |
| GCuC | M | 8 | 6 |  | SD | A | 7 | 6 |
| GFD | P | 14 | 9 |  | SDS | Q | 5 | 5 |
| GFeC | M | 27 | 13 |  | SHS | A | 6 | 4 |
| GFR | P | 7 | 4 |  | SHZnC | M | 2 | 0 |
| GL | A | 2 | 1 |  | SL | A | 31 | 20 |
| GL/GW | A | 5 | 1 |  | SN | A | 18 | 12 |
| GMnC | M | 11 | 6 |  | SNS | A | 38 | 29 |
| GN | A | 4 | 3 |  | SW | A | 10 | 8 |
| GPC | Q | 46 | 32 |  | TKW | A | 105 | 55 |
| GPL | A | 3 | 2 |  | TMRS | P | 7 | 6 |
| GSeC | M | 5 | 3 |  | TN | A | 2 | 2 |
| GW | A | 2 | 0 |  | UIH | A | 1 | 0 |
| GWe | A | 4 | 4 |  | WGC | Q | 1 | 1 |
| GWs | A | 6 | 4 |  | ZnE | M | 2 | 0 |
| † A: Agronomic trait, P: Physiological Trait, M: Micronutrient Trait, Q: Quality Trait.  ‡ Number of initial related individual QTLs to each class of traits which assessed in meta-QTL analysis,  * The full name of assessed traits are displayed in Table 1. | | | | | | | | |
