# Supplementary material for: Comparative Genomic Analysis of Quantitative Trait Loci Associated With Micronutrient Contents, Grain Quality, and Agronomic Traits in Wheat (Triticum aestivum L.)
Source: Front Plant Sci. 2021 Oct 12;12:709817. doi: 10.3389/fpls.2021.709817 (PMC8546302; doi:10.3389/fpls.2021.709817)
Supplement: Supplementary Table 6 — The information of the chi-square test for co-localization frequency analysis of the QTLs of the tested traits in the detected MQTLs regions. [file Table_6.docx]

| **Supplementary Table 6.** Co-localization frequency analysis of the QTLs of the tested traits and the QTLs for GY, GPC, GFeC and GZnC in the detected MQTLs regions in the wheat genome. | | | | | | | | | | | | | | | | | | |  |  |  |  |  |  |  |  |  |  |  |  |  |  |
| --- | --- | --- | --- | --- | --- | --- | --- | --- | --- | --- | --- | --- | --- | --- | --- | --- | --- | --- | --- | --- | --- | --- | --- | --- | --- | --- | --- | --- | --- | --- | --- | --- |
| Traits **^Ϯ^** | Co-localization  with GY | P-Value | Co-localization  with GPC | P-Value | Co-localization  with GFeC | P-Value | Co-localization  with GZnC | P-Value |  | Traits **^Ϯ^** | Co-localization  with GY | P-Value | Co-localization  with GPC | P-Value | Co-localization  with GFeC | P-Value | Co-localization  with GZnC | P-Value |  |  |  |  |  |  |  |  |  |  |  |  |  |  |
| 200KW | 0.0000 | - | 0.0000 | - | 0.0000 | - | 0.0000 | - |  | GY | - | - | 0.4737 | < .00001 | 0.5263 | 0.3009 | 0.4167 | 0.8369 |  |  |  |  |  |  |  |  |  |  |  |  |  |  |
| 25%G | 0.0526 | 0.1549 | 0.0526 | 0.0631 | 0.0000 | 0.4503 | 0.0000 | 0.5485 |  | GZnC | 0.1316 | 0.0245 | 0.2632 | < .00001 | 0.4211 | 0.0002 | - | - |  |  |  |  |  |  |  |  |  |  |  |  |  |  |
| 50%G | 0.1579 | 0.0138 | 0.1053 | 0.0086 | 0.3158 | 0.0003 | 0.1667 | 0.2885 |  | HI | 0.1053 | 0.0443 | 0.2632 | 0.0631 | 0.1053 | 0.2814 | 0.0000 | 0.4386 |  |  |  |  |  |  |  |  |  |  |  |  |  |  |
| 75%G | 0.1579 | 0.0138 | 0.1053 | 0.0086 | 0.1579 | 0.2300 | 0.0833 | 0.9681 |  | HW | 0.1053 | 0.0443 | 0.1579 | 0.0631 | 0.0000 | 0.1483 | 0.0833 | 0.7807 |  |  |  |  |  |  |  |  |  |  |  |  |  |  |
| AGB | 0.0000 | - | 0.0000 | - | 0.0000 | - | 0.0000 | - |  | KH | 0.1316 | 0.0245 | 0.2632 | 0.0002 | 0.3684 | 0.0001 | 0.3333 | 0.0050 |  |  |  |  |  |  |  |  |  |  |  |  |  |  |
| BDT | 0.0263 | 0.3144 | 0.0526 | 0.0631 | 0.0000 | 0.4503 | 0.0000 | 0.5485 |  | KL | 0.0789 | 0.0815 | 0.0526 | < .00001 | 0.0000 | 0.3833 | 0.0000 | 0.4884 |  |  |  |  |  |  |  |  |  |  |  |  |  |  |
| BM | 0.0789 | 0.0815 | 0.1579 | 0.0013 | 0.1053 | 0.1549 | 0.0000 | 0.4884 |  | KW | 0.0526 | 0.1549 | 0.0000 | 0.0002 | 0.0000 | 0.4503 | 0.0000 | 0.5485 |  |  |  |  |  |  |  |  |  |  |  |  |  |  |
| BY | 0.0526 | 0.1549 | 0.0526 | 0.0631 | 0.0000 | 0.5376 | 0.0000 | 0.6242 |  | LDMA | 0.0263 | 0.3144 | 0.0526 | 0.0086 | 0.0000 | 0.3297 | 0.0000 | 0.4386 |  |  |  |  |  |  |  |  |  |  |  |  |  |  |
| CDMA | 0.0000 | - | 0.0526 | 0.0631 | 0.0000 | 0.5376 | 0.0000 | 0.6242 |  | LL | 0.0000 | - | 0.0000 | 0.0086 | 0.0000 | - | 0.0000 | - |  |  |  |  |  |  |  |  |  |  |  |  |  |  |
| CID | 0.0789 | 0.0815 | 0.2632 | 0.0000 | 0.1579 | 0.0354 | 0.2500 | 0.0019 |  | LS | 0.0263 | 0.3144 | 0.0526 | < .00001 | 0.1053 | 0.0582 | 0.0833 | 0.2861 |  |  |  |  |  |  |  |  |  |  |  |  |  |  |
| DA | 0.1053 | 0.0443 | 0.2632 | 0.0000 | 0.2105 | 0.4113 | 0.2500 | 0.3085 |  | LW | 0.0000 | - | 0.0000 | 0.0013 | 0.0000 | - | 0.0000 | - |  |  |  |  |  |  |  |  |  |  |  |  |  |  |
| DDT | 0.0526 | 0.1549 | 0.0000 | - | 0.0000 | 0.2857 | 0.0000 | 0.3961 |  | LY | 0.0526 | 0.1549 | 0.0526 | 0.0631 | 0.0526 | 0.7832 | 0.0000 | 0.4884 |  |  |  |  |  |  |  |  |  |  |  |  |  |  |
| DGC | 0.0263 | 0.3144 | 0.2105 | 0.0002 | 0.1579 | 0.0102 | 0.2500 | 0.0003 |  | MDR | 0.0263 | 0.3144 | 0.0526 | 0.0631 | 0.0000 | 0.4503 | 0.0000 | 0.5485 |  |  |  |  |  |  |  |  |  |  |  |  |  |  |
| DH | 0.1579 | 0.0138 | 0.0000 | - | 0.1579 | 0.2300 | 0.1667 | 0.2885 |  | MRS | 0.1316 | 0.0245 | 0.1053 | 0.009 | 0.1579 | 0.0815 | 0.0833 | 0.7415 |  |  |  |  |  |  |  |  |  |  |  |  |  |  |
| DPM | 0.1316 | 0.0245 | 0.3158 | < .00001 | 0.4211 | 0.0133 | 0.3333 | 0.2106 |  | MTI | 0.0263 | 0.3144 | 0.0000 | - | 0.0000 | 0.3833 | 0.0000 | 0.4884 |  |  |  |  |  |  |  |  |  |  |  |  |  |  |
| DST | 0.0526 | 0.1549 | 0.0526 | 0.0631 | 0.0000 | 0.2857 | 0.0000 | 0.3961 |  | NG | 0.5000 | < .00001 | 0.4737 | < .00001 | 0.4737 | 0.2000 | 0.5000 | 0.2372 |  |  |  |  |  |  |  |  |  |  |  |  |  |  |
| DTF | 0.0263 | 0.3144 | 0.0526 | 0.0631 | 0.0000 | 0.6629 | 0.0000 | 0.7290 |  | PDMA | 0.0000 | - | 0.0526 | 0.0631 | 0.0000 | 0.3297 | 0.0000 | 0.4386 |  |  |  |  |  |  |  |  |  |  |  |  |  |  |
| FFD | 0.0263 | 0.3144 | 0.0000 | - | 0.0000 | 0.6629 | 0.0000 | 0.7290 |  | PGMS | 0.0789 | 0.0815 | 0.0526 | 0.0631 | 0.0000 | 0.2488 | 0.0000 | 0.3594 |  |  |  |  |  |  |  |  |  |  |  |  |  |  |
| FLH | 0.0000 | - | 0.0000 | - | 0.0000 | - | 0.0000 | - |  | PH | 0.2632 | 0.0015 | 0.4211 | < .00001 | 0.4211 | 0.1359 | 0.3333 | 0.5637 |  |  |  |  |  |  |  |  |  |  |  |  |  |  |
| FWA | 0.0263 | 0.3144 | 0.0526 | 0.0631 | 0.0000 | 0.2176 | 0.0000 | 0.3272 |  | PLH | 0.0000 | - | 0.0000 | < .00001 | 0.0000 | - | 0.0000 | - |  |  |  |  |  |  |  |  |  |  |  |  |  |  |
| GAS | 0.0000 | - | 0.0000 | - | 0.0000 | - | 0.0000 | - |  | PT | 0.0526 | 0.1549 | 0.2105 | 0.000 | 0.1579 | 0.0815 | 0.2500 | 0.0072 |  |  |  |  |  |  |  |  |  |  |  |  |  |  |
| GCuC | 0.1053 | 0.0443 | 0.1053 | 0.0086 | 0.2105 | 0.0074 | 0.0000 | 0.3961 |  | SD | 0.1316 | 0.0245 | 0.0000 | - | 0.1053 | 0.9508 | 0.0833 | 0.7807 |  |  |  |  |  |  |  |  |  |  |  |  |  |  |
| GFD | 0.1842 | 0.0078 | 0.2632 | 0.0000 | 0.2105 | 0.3303 | 0.3333 | 0.0508 |  | SDS | 0.1053 | 0.0443 | 0.4737 | < .00001 | 0.1579 | 0.8984 | 0.3333 | 0.1700 |  |  |  |  |  |  |  |  |  |  |  |  |  |  |
| GFeC | 0.2632 | 0.0015 | 0.3684 | < .00001 | - | - | 0.6667 | 0.0002 |  | SHS | 0.0526 | 0.1549 | 0.1053 | 0.009 | 0.0000 | 0.0815 | 0.1667 | 0.1314 |  |  |  |  |  |  |  |  |  |  |  |  |  |  |
| GFR | 0.0789 | 0.0815 | 0.0526 | 0.0631 | 0.1053 | 0.2814 | 0.0833 | 0.6056 |  | SHZnC | 0.0000 | - | 0.0000 | - | 0.2632 | - | 0.0000 | - |  |  |  |  |  |  |  |  |  |  |  |  |  |  |
| GL | 0.0526 | 0.1549 | 0.1053 | 0.0086 | 0.0000 | 0.3833 | 0.1667 | 0.0282 |  | SL | 0.2105 | 0.0044 | 0.2105 | 0.000 | 0.1579 | 0.1514 | 0.2500 | 0.3085 |  |  |  |  |  |  |  |  |  |  |  |  |  |  |
| GL/GW | 0.0789 | 0.0815 | 0.0000 | - | 0.3158 | < .00001 | 0.0833 | 0.7415 |  | SN | 0.2105 | 0.0044 | 0.1053 | 0.009 | 0.4211 | 0.7360 | 0.2500 | 0.2489 |  |  |  |  |  |  |  |  |  |  |  |  |  |  |
| GMnC | 0.0526 | 0.1549 | 0.0526 | 0.0631 | 0.2105 | 0.0074 | 0.0833 | 0.7415 |  | SNS | 0.3421 | 0.0003 | 0.5263 | < .00001 | 0.0000 | 0.2453 | 0.5000 | 0.1498 |  |  |  |  |  |  |  |  |  |  |  |  |  |  |
| GN | 0.0526 | 0.1549 | 0.0526 | 0.0631 | 0.0000 | 0.4503 | 0.0000 | 0.5485 |  | SW | 0.0789 | 0.0815 | 0.1053 | 0.009 | 0.4737 | 0.2488 | 0.0000 | 0.3594 |  |  |  |  |  |  |  |  |  |  |  |  |  |  |
| GPC | 0.2368 | 0.0026 | - | - | 0.3684 | 0.0744 | 0.4167 | 0.0716 |  | TKW | 0.5526 | < .00001 | 0.6316 | < .00001 | 0.2105 | 0.6647 | 0.5833 | 0.3484 |  |  |  |  |  |  |  |  |  |  |  |  |  |  |
| GPL | 0.1316 | 0.0245 | 0.1053 | 0.0086 | 0.3158 | 0.0029 | 0.2500 | 0.1003 |  | TMRS | 0.1579 | 0.0138 | 0.1579 | 0.001 | 0.0000 | 0.0443 | 0.2500 | 0.0373 |  |  |  |  |  |  |  |  |  |  |  |  |  |  |
| GSeC | 0.0000 | - | 0.0000 | - | 0.0526 | 0.3145 | 0.0833 | 0.1208 |  | TN | 0.0263 | 0.3144 | 0.0526 | 0.0631 | 0.0000 | 0.6629 | 0.0000 | 0.7290 |  |  |  |  |  |  |  |  |  |  |  |  |  |  |
| GW | 0.0263 | 0.3144 | 0.0000 | - | 0.0000 | 0.6629 | 0.0000 | 0.7290 |  | UIH | 0.0000 | - | 0.0000 | < .00001 | 0.0000 | - | 0.0000 | - |  |  |  |  |  |  |  |  |  |  |  |  |  |  |
| GWe | 0.0263 | 0.3144 | 0.0000 | - | 0.0526 | 0.5690 | 0.0000 | 0.5485 |  | WGC | 0.0263 | 0.3144 | 0.0526 | 0.0631 | 0.0000 | 0.6629 | 0.0000 | 0.7290 |  |  |  |  |  |  |  |  |  |  |  |  |  |  |
| GWs | 0.0789 | 0.0815 | 0.0526 | 0.0631 | 0.0526 | 0.5690 | 0.0000 | 0.5485 |  | ZnE | 0.0000 | - | 0.0000 | - | 0.4211 | - | 0.0000 | - |  |  |  |  |  |  |  |  |  |  |  |  |  |  |
| ^Ϯ^ The full name of assessed traits are displayed in Table 1. | | | | | | | | | | | | | | | | | | |  |  |  |  |  |  |  |  |  |  |  |  |  |  |
|  | | | | | | | | | | | | | | | | | | |  |  |  |  |  |  |  |  |  |  |  |  |  |  |
